# Histone Deacetylase HDA9 With ABI4 Contributes to Abscisic Acid Homeostasis in Drought Stress Response

**DOI:** 10.3389/fpls.2020.00143

**Published:** 2020-02-25

**Authors:** Dongwon Baek, Gilok Shin, Min Chul Kim, Mingzhe Shen, Sang Yeol Lee, Dae-Jin Yun

**Affiliations:** ^1^Division of Applied Life Science (BK21plus program), Plant Molecular Biology and Biotechnology Research Center, Gyeongsang National University, Jinju, South Korea; ^2^Department of Biomedical Science and Engineering, Konkuk University, Seoul, South Korea; ^3^Institute of Agriculture & Life Science, Gyeongsang National University, Jinju, South Korea

**Keywords:** drought stress, abscisic acid, histone deacetylase, HDA9, ABI4, 8′-hydroxylase, *Arabidopsis thaliana*

## Abstract

Drought stress, a major environmental factor, significantly affects plant growth and reproduction. Plants have evolved complex molecular mechanisms to tolerate drought stress. In this study, we investigated the function of the *Arabidopsis thaliana* RPD3-type HISTONE DEACETYLASE 9 (HDA9) in response to drought stress. The loss-of-function mutants *hda9-1* and *hda9-2* were insensitive to abscisic acid (ABA) and sensitive to drought stress. The ABA content in the *hda9-1* mutant was reduced in wild type (WT) plant. Most histone deacetylases in animals and plants form complexes with other chromatin-remodeling components, such as transcription factors. In this study, we found that HDA9 interacts with the ABA INSENSITIVE 4 (ABI4) transcription factor using a yeast two-hybrid assay and coimmunoprecipitation. The expression of *CYP707A1* and *CYP707A2*, which encode (+)-ABA 8′-hydroxylases, key enzymes in ABA catabolic pathways, was highly induced in *hda9-1*, *hda9-2*, *abi4*, and *hda9-1 abi4* mutants upon drought stress. Chromatin immunoprecipitation and quantitative PCR showed that the HDA9 and ABI4 complex repressed the expression of *CYP707A1* and *CYP707A2* by directly binding to their promoters in response to drought stress. Taken together, these data suggest that HDA9 and ABI4 form a repressive complex to regulate the expression of *CYP707A1* and *CYP707A2* in response to drought stress in *Arabidopsis*.

## Introduction

Drought stress causes serious damage to plant growth, survival, and productivity (Zhu, 2001). Plants have evolved a variety of complex signaling mechanisms to sense and acclimate to drought stress (Bohnert and Sheveleva, 1998). Accumulation of the phytohormone, abscisic acid (ABA), helps to protect plants from drought stress by controlling seed maturation and dormancy during seed development and seedling growth (Finkelstein and Lynch, 2000; Lopez-Molina et al., 2001). ABA also plays a major role in regulating physiological processes in the drought stress signaling pathway, primarily by controlling stomatal aperture and modulating the expression of many ABA-responsive genes that function in drought tolerance (Yamaguchi-Shinozaki and Shinozaki, 2006; Cutler et al., 2010; Raghavendra et al., 2010).

The levels of endogenous ABA are regulated by the balance of ABA biosynthesis and catabolism (Xiong and Zhu, 2003; Nambara and Marion-Poll, 2005). When plants are exposed to drought stress or exogenous ABA, most of the major enzymes of the ABA biosynthesis pathway, including a zeaxanthin epoxidase (ZEP/AtABA1), 9-*cis*-epoxycarotenoid dioxygenase3 (NCED3), molybdenum cofactor (moco/AtABA3), and *Arabidopsis* aldehyde oxidase3 (AAO3), are activated, with the exception of short-chain dehydrogenase/reductase (SDR/AtABA2) (Xiong and Zhu, 2003; Dong et al., 2015). In ABA catabolism, endogenous ABA is converted from the active to the inactive form through degradation to generate phaseic acid (PA) by ABA 8′-hydrolases, a key enzyme in ABA catabolic pathways (Kushiro et al., 2004; Dong et al., 2015). During seed imbibition, reduction of ABA levels is associated with increasing levels of PA in plants (Jacobsen et al., 2002; Kushiro et al., 2004). The ABA 8′-hydroxylase family includes four members, CYP707A1 to CYP707A4, which increase in abundance during seed dormancy and germination (Jacobsen et al., 2002; Kushiro et al., 2004). CYP707A1 and CYP707A2 cause a sharp reduction in ABA levels during seed maturation and germination (Jacobsen et al., 2002; Okamoto et al., 2006). However, CYP707A4 diurnally regulates ABA levels through conjugation/deconjugation (Pan et al., 2009). In plants, large amounts of ABA are converted into the ABA-glucose ester, the inactive glucose conjugate form, and are transported to the vacuoles by the *β*-glucosidase AtBG1 (Lee et al., 2006).

ABA signaling pathways for seed maturation and dormancy are controlled by ABA-responsive transcription factors that regulate gene expression (Finkelstein et al., 2002). The representative transcription factors (*e.g.*, ABA INSENSITIVE 3 (ABI3), ABI4, and ABI5) are associated with regulating sensitivity to ABA (Finkelstein et al., 2002; Shu et al., 2016). In addition, ABI3, ABI4, and ABI5 have distinct roles in ABA-dependent seed maturation and dormancy (Shu et al., 2016). ABI3 is a negative regulator of seed germination in both the ABA and gibberellic acid signaling pathways (Yuan and Wysocka-Diller, 2006). Although ABI5 interacts with ABI3, ABI5 is epistatic to ABI3 (Lopez-Molina et al., 2002). ABI5 affects the ubiquitin ligase activity of KEEP ON GOING (KEG) following germination (Stone et al., 2006). ABI4, which is specifically expressed in the embryo, plays a unique and important role in the catabolism of embryonic lipids during seed germination (Penfield et al., 2006).

Epigenetic modifications are important regulatory mechanisms of gene transcription and play essential roles in plant development and stress responses (Yuan et al., 2013). The reversible process of histone acetylation and deacetylation by histone acetyltransferases (HATs) and histone deacetylases (HDACs) has been implicated in genome expression, chromatin structural organization, and gene function (Wang et al., 2014; Verdin and Ott, 2015). The HDAC family in *Arabidopsis* is made up of 18 members and is categorized into three groups: twelve REDUCED POTASSIUM DEPENDENCY PROTEIN 3 (RPD3/HDA1), four HISTONE DEACETYLASE 2 (HD2), and two SILENT INFORMATION REGULATOR PROTEIN 2 (SIR2) (Pandey et al., 2002; Hollender and Liu, 2008). HDACs in the RPD3/HDA1 group have multiple functions, including plant development, DNA methylation, and pathogen defense signaling (Aufsatz et al., 2002; Zhou et al., 2005; Liu et al., 2013; Cigliano et al., 2013; Ryu et al., 2014). HD2 HDACs are plant-specific and are involved in developmental processes and stress responses (Zhou et al., 2004; Sridha and Wu, 2006; Luo et al., 2012a). HDACs in the SIR2 group are associated with energy metabolism in the mitochondria and the transition of leaf tissue to callus (König et al., 2014; Lee et al., 2016).

The RPD3 group is further classified into three classes based on sequence similarity and phylogenetic analysis, with four HDACs belonging to class I (HDA19, HDA6, HDA7, and HDA9), three to class II (HDA5, HDA15, and HDA18), and one to class III (HDA2). HDA8, HDA14, HDA10, and HDA17 of the RPD3 group are unclassified members (Pandey et al., 2002; Hollender and Liu, 2008). Recent studies have suggested that the HDACs in class I are involved in abiotic stress signaling, responding to salt, ABA, and drought stress (Song et al., 2005; Sridha and Wu, 2006; Chen and Wu, 2010; Luo et al., 2012a; Ryu et al., 2014). HDA6 and HDA19 play an important role in modulating seed germination during salt stress responses and in response to ABA, as well as abiotic stress-induced gene expression in *Arabidopsis* (Chen and Wu, 2010; Luo et al., 2012b; Ryu et al., 2014). Furthermore, HDA19 acts as a transcriptional repressor through the formation of a protein complex with AtERF7 and AtSin3 (Song et al., 2005). In contrast to the *hda19* and *hda6* mutants, the HDA9 mutants, *hda9-1* and *hda9-2*, were insensitive to salt stress and ABA during seedling root growth and seed germination (Zheng et al., 2016). Moreover, HDA9 forms a complex with PWR and transcription factor WRKY53, and PWR plays an important role as a regulator for HDA9 nuclear accumulation (Chen et al., 2016). Several class I HDACs function as positive regulators of stress responses; however, a few others negatively regulate plant stress responses by repressing the expression of stress-responsive genes (Zheng et al., 2016).

Although the function of HDA9 in salt and drought stress responses can be explained by its negative effects on the expression of stress-responsive genes, the mechanism by which it affects other aspects of stress signaling pathways remains unknown. Here, we show that HDA9 acts in association with an ABA-related transcription factor to repress gene expression through histone deacetylation. HDA9 interacts with ABI4 *in vivo*, and the HDA9–ABI4 complex acts together on *AtCYP707A1* and *AtCYP707A2* in the regulation of drought stress. Furthermore, the HDA9-ABI4 complex regulates the levels of endogenous ABA during drought stress. These findings revealed that the HDA9–ABI4 complex regulates histone deacetylation to control a wide variety of biological processes during drought stress responses.

## Materials and Methods

### Plant Materials and Growth Conditions

The wild type (WT) *Arabidopsis thaliana* used in this study was Col-0 ecotype, and all mutants were on the Col-0 background. Seeds were grown on 1/2 Murashige and Skoog (MS) media with 1.5% (w/v) sucrose, pH 5.7 under a long-day photoperiod (16 h light/8 h dark) at 23°C. The *hda9-1* (SALK_007123) mutant was obtained from NASC (http://arabidopsis.info/), and *hda9-2* (Gk_305G03) mutant from ABRC (http://www.arabidopsis.org/) (van Zanten et al., 2014). Dr. Nho kindly provided two seeds of *hda9-1*/HDA9 complementation plant (#7 and #11 lines) with a C-terminal HA tag (Kang et al., 2015).

We generated the *hda9-1abi4* double mutant by crossing the homozygous *hda9-1* and *abi4* (CS8104; Zhang et al., 2013) single mutant and then self-pollinated the F1 generation. To select the compatible lines of F2 (10 lines) and homozygous F3 (5 lines) generation, we performed PCR genotyping analysis and DNA sequencing (**Supplemental Table S2**).

### Physiological Assay

For ABA germination assays, seeds were grown on 1/2 MS medium containing 1.5% sucrose with various concentrations of ABA (Sigma, St. Louis, MO, USA). Successful germination in the presence of ABA was determined by the presence of green cotyledons 5 d after sowing. Four experimental repeats were carried out, each one containing at least 32 seeds. For drought tests, one-week-old seedlings grown on MS medium were transferred to the soil. Transferred seedlings were adapted to the soil for one week under identical conditions followed by withholding water for 11 d. After rewatering for 1 d, recovery of WT, mutants and transgenic plants was monitored. Three experimental repeats were carried out each one containing at least 36 plants. For water loss assays, one-week-old seedlings grown on MS medium were transferred to the soil. After growing for two weeks, plant shoots were cut and placed in petri dishes. The dishes were maintained in the growth chamber and the loss of fresh weight was determined at the indicated times. The experiments were performed with three independent replicates with eight plants per replicate.

### Stomatal Aperture Bioassays

Developmentally similar leaves were detached from 10-day-old seedlings and floated on stomatal opening buffer (5 mM MES, 5 mM KCl, 50 µM CaCl_2_, pH 5.6) under light for 2 h and then treated with 5 µM ABA for 2 h. After ABA treatment, the leaves were fragmented using a scalpel, and the epidermal fragments were quickly mounted for visualization in a scanning electron microscope (JSM-6380LV; JEOL, Akishima, Japan). The stomatal aperture was determined from measurements of 50 to 80 stomata per treatment. Each experiment was repeated four times.

### Yeast Two-Hybrid Assay

For yeast two-hybrid experiments, *pDONR*™*/Zeo*-*HDA9* and *pDONR*™*/Zeo*-*ABI4* were fused in the yeast two-hybrid destination vector *pDEST22* (harboring activation domain) and *pDEST32* (harboring DNA binding domain) to generate construct vector, *pDEST22-HDA9* and *pDEST32-ABI4*, respectively. These plasmids were transformed into the *Saccharomyces cerevisiae* (*YRG2*). Protein–protein interactions were determined by the growth of yeast colonies on SD/-Trp-Leu (Sc-TL) or SD/-Trp-Leu-His (Sc-TLH; Takara Bio, Kusatsu, Japan) agar media containing 3-amino-1,2,4-triazole (3-AT; 25 mM). Empty vector was used as a negative control.

### Protein Extraction and CoImmunoprecipitation Assay

Total protein was extracted from three-week-old *Nicotiana benthamiana* leaves and the extraction buffer contained 100 mM Tris-Cl, pH 7.5, 150 mM NaCl, 0.5% NP-40, 1 mM EDTA, 3 mM DTT and protease inhibitors (1 mM PMSF, 5 μg ml^−1^ leupeptin, 1 μg ml^−^ aprotinin, 1 μg/ml pepstatin, 5 μg/ml antipain, 5 μg/ml chymostatin, 2 mM Na_2_VO_3_, 2 mM NaF, and 50 mM MG132) (Park et al., 2018). For coimmunoprecipitation assays of *N. benthamiana*, leaves were coinfiltrated with *Agrobacterium tumefaciens* (*GV3101*) cell expressing the indicated plasmid combination using *35S:HDA9-3xHA* and *35S:ABI4-GFP*. Total protein reacted for immunoprecipitation using rabbit anti-GFP polyclonal (Abcam, Cambridge, MA, USA) and protein A agarose (Invitrogen, Carlsbad, CA, USA). For immunoblotting, membranes were incubated with the appropriate anti-HA (Roche, Indianapolis, IN, USA), and detected using ECL-detection reagent (GE Healthcare, Little Chalfont, Buckinghamshire, UK). For the coimmunoprecipitation assays three independent replicates were carried out.

### RNA Isolation and Quantitative PCR Analyses

Total RNA was extracted from plants (harvest timing is described in each experiment) with the RNeasy Plant Mini Kit (Qiagen, Hilden, Germany) and treated with DNase (Sigma, St. Louis, MO, USA). 2 µg RNAs were used for the synthesis of the first-strand cDNA using the Thermoscript™ RT-PCR System (Invitrogen, Carlsbad, CA, USA). Quantitative PCR was performed using SYBR Green PCR Master Mix kit (SYBR Green Supermix; Bio-Rad, Hercules, CA, USA) according to instructions with the CFX96 real-time PCR detection system (Bio-Rad, Hercules, CA, USA). The expression of *TUBULIN8* was used as the endogenous control. The qRT-PCR experiments were performed in three independent replicates. The sequences of primers used in qRT-PCR are listed in **Supplemental Table S2**.

### Quantitative Determination of Abscisic Acid

Endogenous ABA was extracted from 10-day-old plants with/without dehydration stress and analyzed using Phytodetek ABA test Kit (Agdia Incorporated, IN, USA) according to the manufacturer's instruction. Three biological repeats and three technical repeats were performed and measured for each sample.

### Chromatin Immunoprecipitation Assay

The ChIP assays were performed as previously described (Saleh et al., 2008). Nuclei from two-week-old *hda9-1HDA9-HA* plants were extracted with CelLytic™ PN Isolation/extraction Kit (Sigma, St. Louis, MO, USA) and sonicated (Bioruptor, Tokyo, Japan). Immunoprecipitations were carried out using an anti-HA antibody (Roche, Indianapolis, IN, USA) with salmon sperm DNA/protein A agarose (Upstate, New York, USA) beads. Treatment with antirat IgG was used as a negative control to detect background levels in each ChIP experiment. The immunoprecipitated DNA was quantified by qRT-PCR analysis. The expression of *TUBULIN4* was used as the internal control. The ChIP experiments were performed in three independent replicates. The specific primer sequences are provided in **Supplemental Table S2**.

## Results

### HDA9 Mediates in Seed Germination and Stomatal Closure by ABA

**Figure 1:**
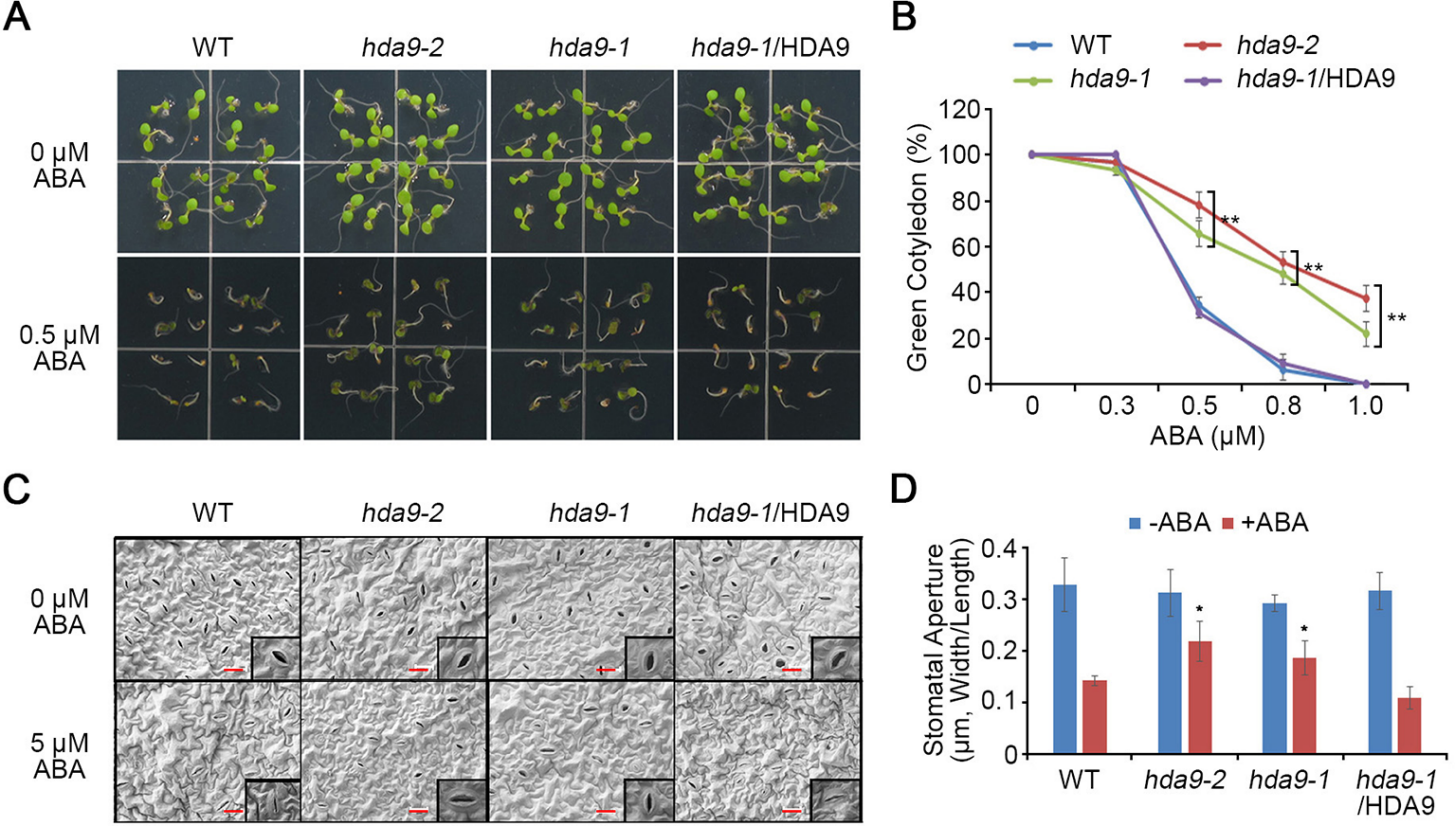
*hda9* mutants are insensitive to ABA. **(A)** Seeds of WT, *hda9-1*, *hda9-2*, and *hda9-1*/HDA9 were germinated on MS media supplemented with and without 5 μM ABA. Photographs were taken 5 d after sowing. **(B)** Quantification of germination rate of WT, *hda9-1*, *hda9-2*, and *hda9-1*/HDA9 on different concentrations of ABA. The values indicated means ± SE of n = 4 biological replicates of at least 32 seeds for each experiment. Asterisks represent significant differences from the WT (*, 0.01 < *p*-value ≤ 0.05; **, *p*-value < 0.01; Student's *t*-test). **(C)** ABA-induced stomata closure. Leaves of 10-d-old seedling of WT, *hda9-1*, *hda9-2*, and *hda9-1*/HDA9 were floated in stomatal opening solution for 2 h and then incubated in the 5 μM ABA for 2 h in the light. Stomata on the abaxial surface were observed using scanning electron microscopy. **(D)** Measurement of stomatal aperture (the ratio of width to length) after ABA treatment. The values indicated means ± SE of n = 4 biological replicates of 50 to 80 stomata for each experiment. Asterisks represent significant differences from the WT (*, 0.01 < p-value ≤ 0.05; Student's *t*-test).

To investigate functional characterization of HDA9, two independent mutants, *hda9-1* (SALK_007123) and *hda9-2* (CS370750), were obtained from the *Arabidopsis* Biological Resource Center (**Supplementary Figure S1**). The *hda9-*1 and *hda9-2* mutants have T-DNA insertions in the first intron and fifth exon of HDA9, respectively (**Supplementary Figure S1A**). The presence of the T-DNA at the expected location in the *hda9* mutants was verified by genome diagnostic PCR (**Supplementary Figure S1B**). Quantitative reverse transcription PCR (qRT-PCR) analyses indicated that *hda9-1* and *hda9-2* were RNA null mutants (**Supplementary Figure S1C**). Although HDA9 is involved in seed dormancy and germination, the ABA sensitivity of *hda9* mutant is similar compared to that of WT (van Zanten et al., 2014). ABA enhances seed dormancy and inhibits germination and root growth by regulating the balance of endogenous phytohormones (Finkelstein et al., 2008). To investigate the function of HDA9 in ABA signaling, we examined the physiological responses of the *hda9* mutants (*hda9-1* and *hda9-2*), and *HDA9 promoter:HDA9-HA* in the *hda9-1* mutant (#11 line; *hda9-1*/HDA9) complemented transgenic plants in response to ABA. Compared to the WT plants, seed germination of the *hda9* mutants was significantly enhanced when exposed to exogenous ABA. However, the *hda9-1*/HDA9 plant showed a similar phenotype to the WT plants (**Figure 1A**). The percentage of fully opened green cotyledons in WT, *hda9* mutants, and *hda9-1*/HDA9 were similar in the absence of ABA (**Figure 1B**). However, in the presence of 0.5 µM ABA, the percentage of green cotyledons in the *hda9* mutants were approximately 70–80%, compared to 35–40% for the WT and the *hda9-1*/HDA9 seedlings (**Figure 1B**).

Upon sensing abiotic stress, plants increase their endogenous ABA content to induce stomatal closure (Vishwakarma et al., 2017). To investigate whether HDA9 influences ABA-mediated changes in the stomata, stomatal closure responses were determined in the WT, *hda9* mutants, and *hda9-1*/HDA9 plants under exogenous ABA treatment. The guard cell sizes, stomatal density, and stomatal apertures were similar in the leaves of the WT, *hda9* mutants, and *hda9-1*/HDA9 plants at the same developmental stage (**Figures 1C, D**). However, the stomata closing in *hda9* mutants was delayed in response to ABA compared to those in WT and the *hda9-1*/HDA9 plants (**Figures 1C, D**). Therefore, these results suggested that HDA9 plays a critical role in regulating ABA-dependent stomatal closure and ABA sensitivity during seed germination.

### HDA9 Regulates ABA-Dependent Hypersensitivity to Drought Stress

**Figure 2:**
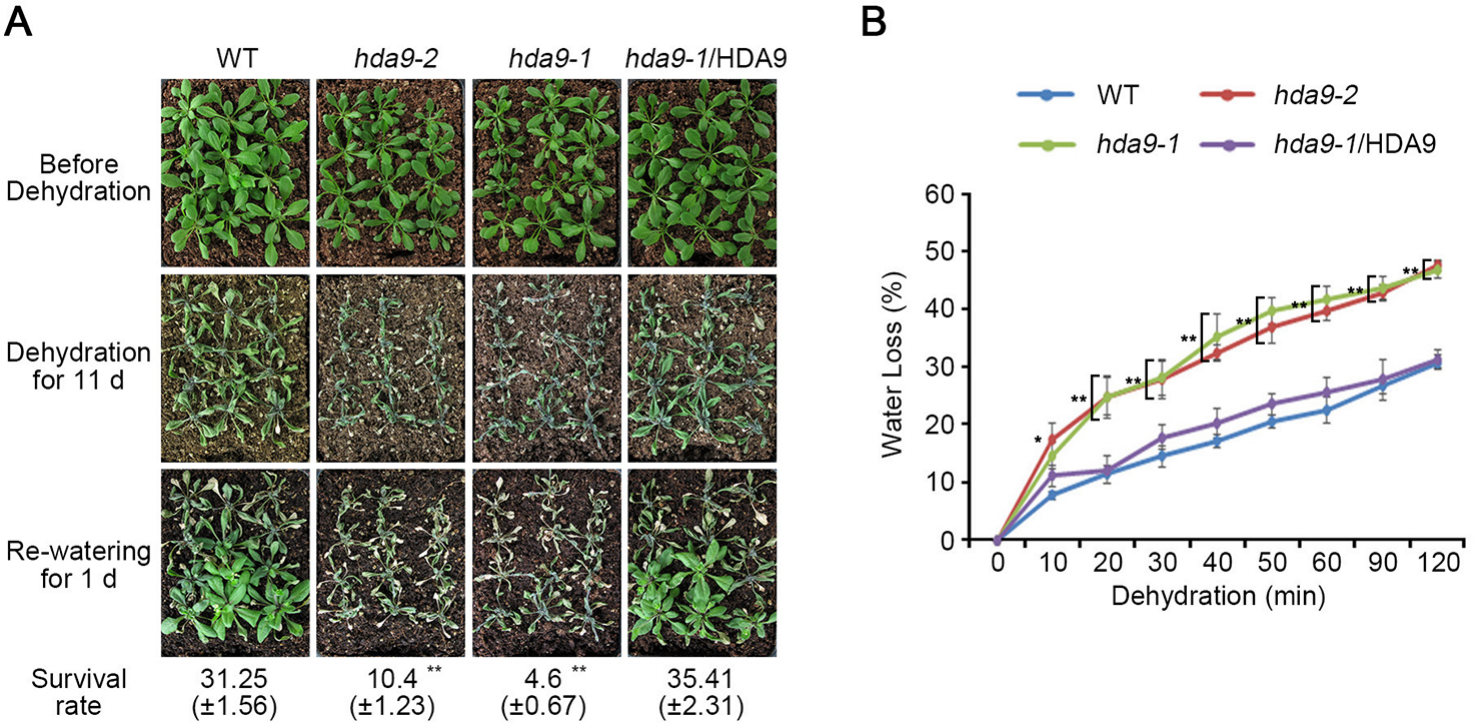
*hda9* mutants are sensitive to dehydration. **(A)** One-week-old plants were transferred to soil and grown for an additional one week. Photographs of plants before and after stress treatment. Water was withheld from two-week-old plants for 11 d, and then plants were rewatered for 1 d before the photograph was taken. Quantitation of the survival rate of WT, *hda9-1*, *hda9-2* and *hda9-1*/HDA9 plants. The values of survival rate indicated means ± SE of n = 3 biological replicates of at least 36 plants for each experiment. **(B)** Water loss assay. Water loss is presented as the percentage of weight loss versus initial fresh weight from three-week-old WT, *hda9-1*, *hda9-2* and *hda9-1*/HDA9 plants. Water loss was calculated from the results of three independent experiments. The values of survival rate indicated means ± SE of n = 3 biological replicates of at least 8 plants for each experiment. Asterisks represent significant differences from the WT (*, 0.01 < *p*-value ≤ 0.05; **, *p*-value < 0.01; Student's *t*-test).

Stress phytohormone ABA quickly accumulates in plant tissues that are exposed to drought stress (Xu et al., 2013). Increased endogenous ABA leads to stomatal closure to minimize water loss through transpiration (Blatt, 2000). As ABA-induced stomata closing was partially suppressed in *hda9* mutants, we compared the drought tolerance of the WT, the *hda9* mutants, and *hda9-1*/HDA9 plants by counting the number of plants that survived following water deficit (**Figure 2**). Three-week-old WT, *hda9* mutants, and *hda9-1*/HDA9 plants were deprived of water for 11 d. One day after rewatering, approximately 30% of the WT and *hda9-1*/HDA9 plants survived, whereas only 5% of the *hda9* mutants survived (**Figure 2A**). In addition, the *hda9* mutants showed wilted and purple tint leaves (**Figure 2A**). The water loss in the *hda9* mutant leaves was approximately 25% more rapid than in the WT plants as measured by the progressive water loss from detached leaves (**Figure 2B**). Also, the transcriptional expression of *HDA9* was weakly induced under drought stress condition (**Supplemental Figure S2**). These results demonstrated that HDA9 plays a positive role in plant responses to drought stress.

### HDA9 Interacts With the ABI4 Transcription Factor *In Vivo*

**Figure 3:**
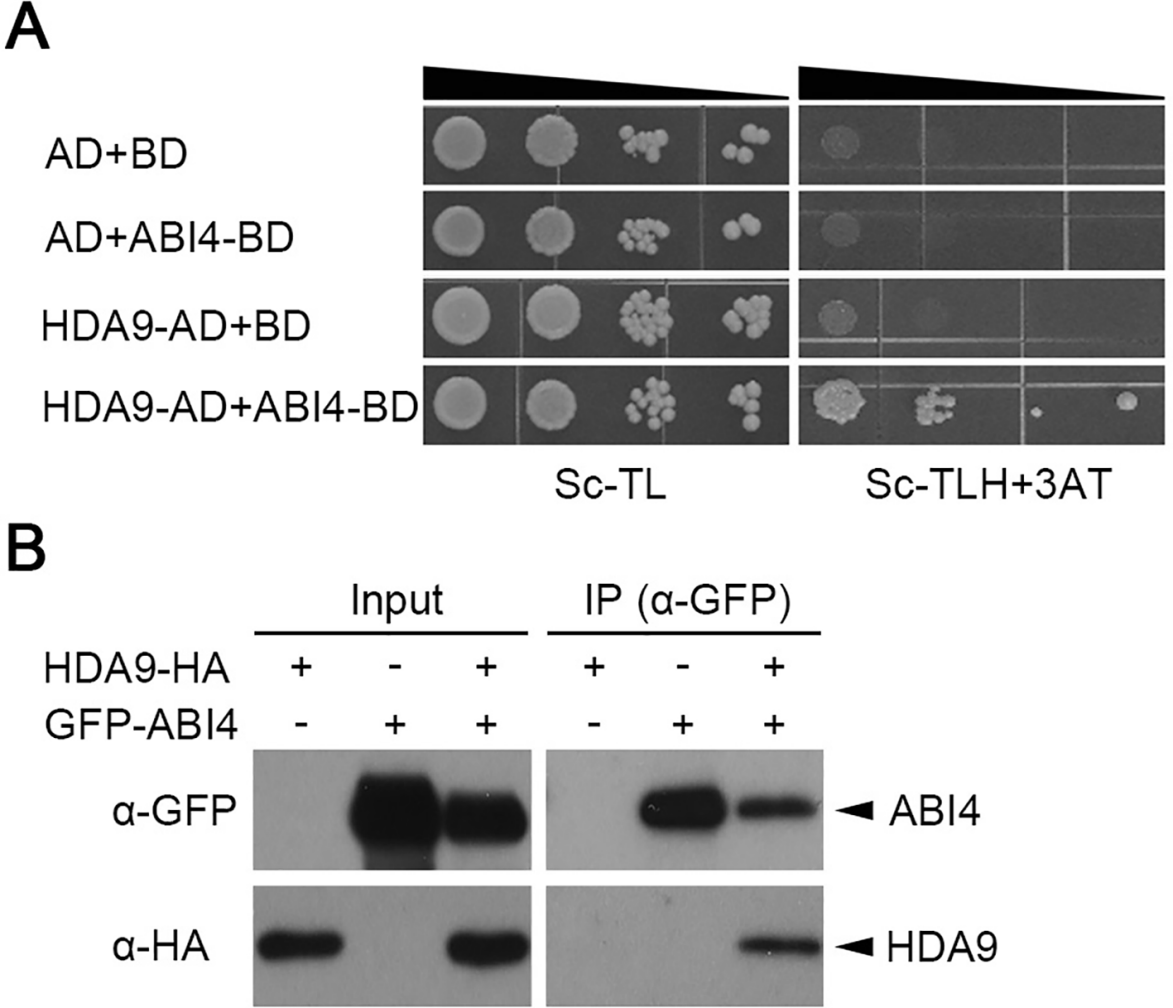
HDA9 interacts with ABI4 *in vivo*. **(A)** Interaction between HDA9 and ABI4 by yeast two hybrid assay. BD, *pDEST32* is the bait plasmid; AD, *pDEST22* the prey plasmid. The cotransformed yeast strains were plated on the control SD-TL and selective medium SD-TLH plus 25 mM 3-AT. The combinations with empty plasmid were used as negative controls. **(B)** Coimmunoprecipitation assay between HDA9 and ABI4 proteins. Protein extracts obtained from tobacco leaves infiltrated with *Agrobacterium* harboring *35S::HDA9-HA* and *35S::ABI4-GFP* were analyzed using anti-GFP and anti-HA antibodies. Input levels of epitope tagged proteins in crude protein extracts were analyzed by immunoblotting. Immunoprecipitated epitope GFP-tagged proteins were probed with anti-HA antibodies to detect coimmunoprecipitation of ABI4-GFP with HDA9-HA.

HDACs are known to interact with DNA-binding proteins to modulate gene transcription in abiotic stress responses (Song et al., 2005; Song and Galbraith, 2006). ABI3, ABI4, and ABI5 are important regulatory transcription factors during seed dormancy, germination, and development (Lopez-Molina et al., 2001; Nambara and Marion-Poll, 2005; Shu et al., 2013). To identify the mode of HDA9 function during seed germination, we tested its interaction with several known ABA-mediated transcription factors, including ABI3, ABI4, and ABI5, using the yeast two-hybrid system. Full-length cDNAs of ABI3, ABI4, and ABI5 were fused to the GAL4 activation domain and HDA9 was used as bait. The empty vectors were used as a negative control. The yeast two-hybrid assay showed that HDA9 interacts with ABI4 but not with ABI5 (**Figure 3A** and **Supplementary Figure S3**). However, HDA9 did not interact with other transcription factors, which were related in ABA responses, such as ABF1, ABF2, and ABF3 (**Supplementary Figure S3**). Also, it is not clear whether HDA9 interacts with ABI3 and/or ABF4 because of auto-activation activities of ABI3 and ABF4 in the yeast two-hybrid system using *pDEST22* and *pDEST32* vector (**Supplementary Figure S3**). To confirm the interaction of HDA9 with ABI4, we performed coimmunoprecipitation assays by using the C-terminal HA-tagged HDA9 (HDA9-HA) and the N-terminal GFP-tagged ABI4 (GFP-ABI4). Briefly, we transiently coexpressed HDA9-HA and GFP-ABI4 in *N*. *benthamiana* leaves using agro-infiltration. We used anti-GFP antibodies to immunoprecipitate GFP-ABI4 and performed western blots with anti-HA antibodies for analysis of the HDA9 immunocomplexes (**Figure 3B**). These results indicate that HDA9 binds specifically to ABI4 transcription factor *in vivo*.

### HDA9 Alters the Expression of ABA Catabolism-Related Genes in Drought Stress Response

**Figure 4:**
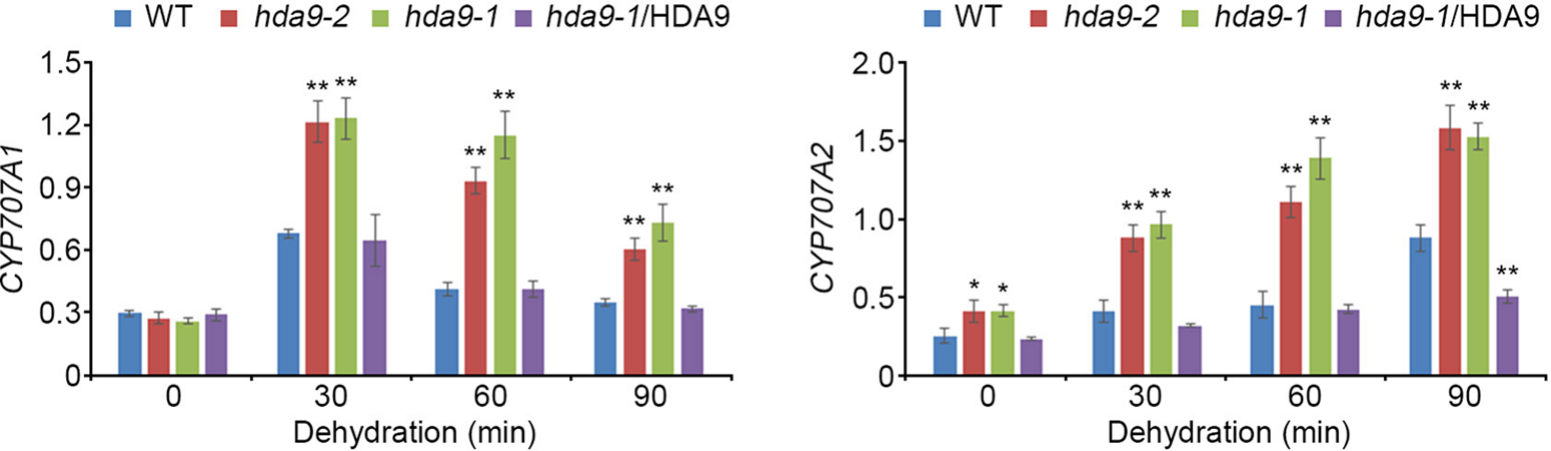
HDA9 regulates *CYP707A1* and *CYP707A2* expression to dehydration. Quantitative RT-PCR analyses of *CYP707A1* and *CYP707A2* in WT, *hda9-1*, *hda9-2* and *hda9-1*/HDA9 after dehydration stress treatment. Total RNA was extracted from 10-d-old seedlings treated with dehydration stress for indicated times. Expression of *TUBULIN8* was used for normalization. Bars represent mean ± SD of three biological replicates with three technical replicates each. Asterisks represent significant differences from the WT (*, 0.01 < *p*-value ≤ 0.05; **, *p*-value < 0.01; Student's *t*-test).

**Figure 5:**
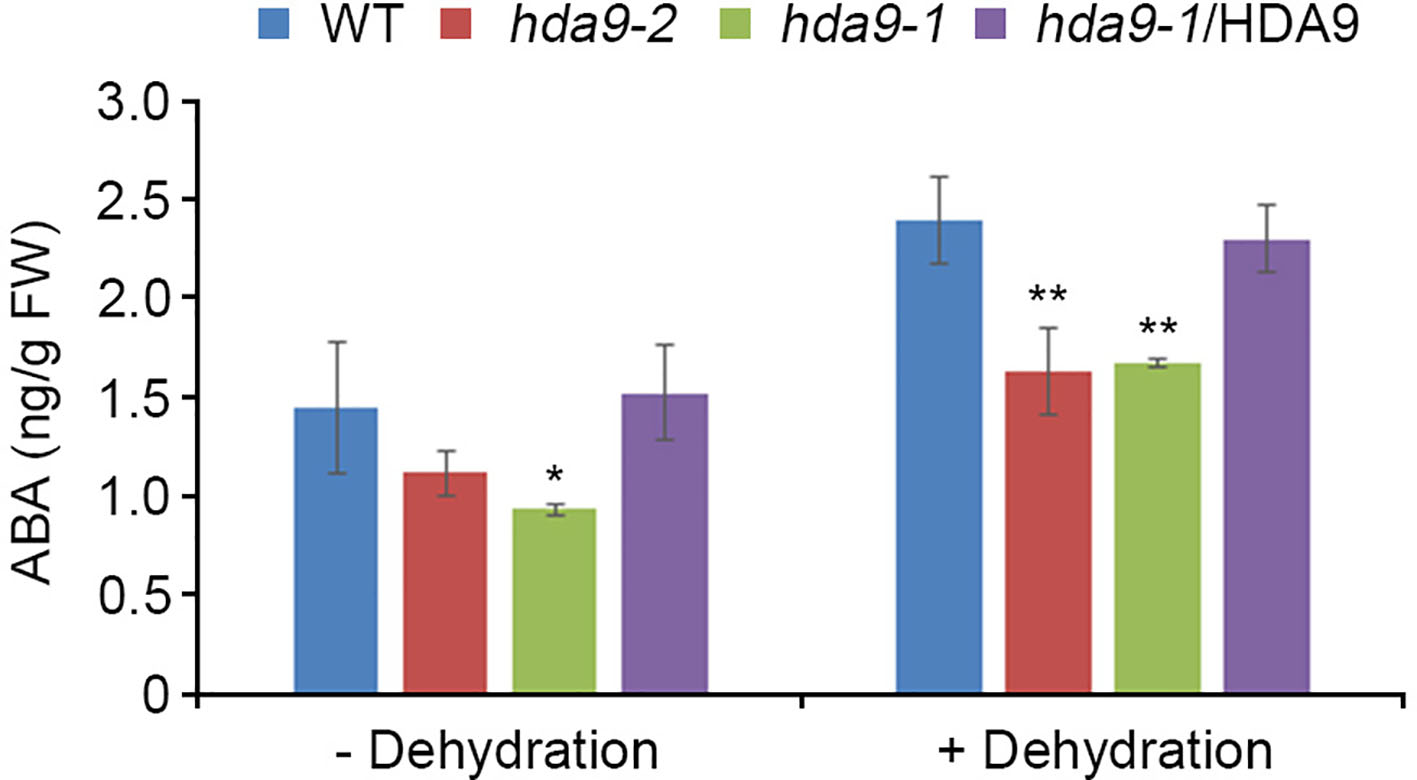
HDA9 regulates intracellular ABA levels under dehydration stress. ABA content in detached 10-d-old WT, *hda9-1*, *hda9-2*, and *hda9-1*/HDA9 plants during a 1-h dehydration. ABA content was measured from 20 whole seedlings of each genotype. Error bars represent the SD from four independent experiments. Asterisks represent significant differences from the WT (*, *p*-value ≤ 0.05, **, *p*-value ≤ 0.01, Student's *t*-test).

ABI4, an AP2/ERF transcription factor, acts both as a positive and a negative regulator in ABA signal transduction during seed dormancy and germination (Finkelstein and Rock, 2002). To investigate the regulatory functions of HDA9 in drought stress response, we tested the transcript levels of six genes, *Lhcb1.2*, *AOX1a*, *CYP707A1*, *CYP707A2*, *ACS4*, and *ACS8*, which were negatively regulated by ABI4, in the WT, *hda9-1* mutant, and the *hda9-1*/HDA9 plants under drought stress conditions (Koussevitzky et al., 2007; Giraud et al., 2009; Shu et al., 2013; Dong et al., 2016; **Supplementary Table S1**). The transcripts of *CYP707A1* and *CYP707A2* were significantly up-regulated in the *hda9-1* and *hda9-2* mutants compared to those in the WT under drought stress conditions, whereas the *hda9-1*/HDA9 plant showed a similar expression patterns of *CYP707A1* and *CYP707A2* to the WT (**Figure 4**). However, the transcripts of *Lhcb1.2*, *AOX1a*, *ACS4*, and *ACS8* were not affected by drought stress in the WT, *hda9-1* mutant, and *hda9-1*/HDA9 plants (**Supplemental Figure S4**). In addition, because *CYP707A1* and *CYP707A2* played important roles in ABA catabolism, we measured ABA content in the WT, *hda9-1* mutant, and *hda9-1*/HDA9 plants under drought stress conditions. The ABA content in the *hda9-1* mutant seedlings was approximately 1.5-fold lower than that in the WT and *hda9-1*/HDA9 seedlings (**Figure 5**), and the ABA content in seeds of *hda9* mutant was lower than that in seeds of WT (**Supplemental Figure S5**). These results confirmed that HDA9 negatively regulated transcriptional expression of ABA catabolism-related genes for maintaining ABA level in drought stress response.

### HDA9 Associated With *CYP707A1* and *CYP707A2* Promoters

**Figure 6:**
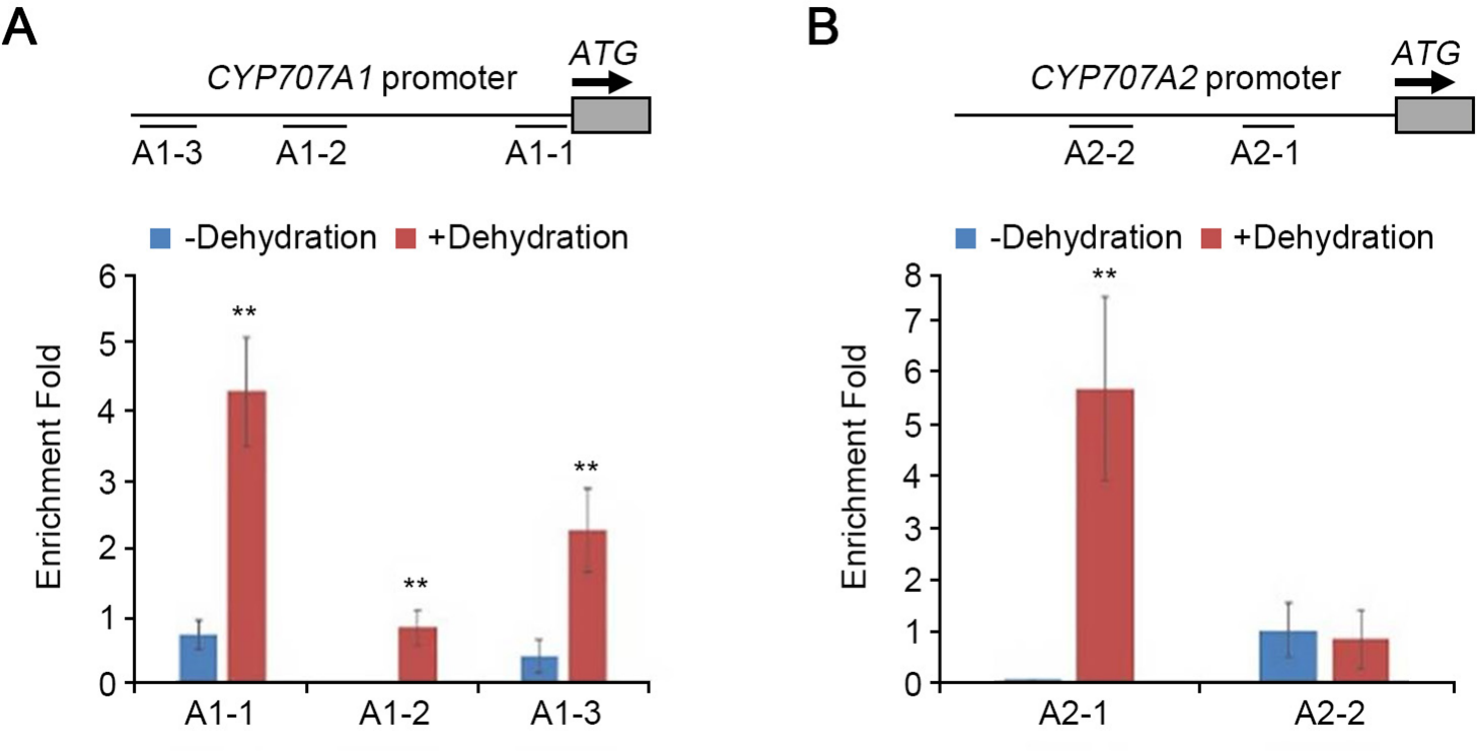
HDA9 is associated with the promoters of *CYP707A1* and *CYP707A2* in dehydration response. The ChIP assays of the *CYP707A1* and *CYP707A2* chromatin regions associated with HDA9. The ChIP assays were performed on nuclear proteins extracted from two-week-old seedling *hda9-1*/HDA9 (with HA tag) plants with/without dehydration stress. Chromatin complex was immunoprecipitated with anti-HA antibody. Samples were quantified by real-time qPCR using specific primers for the amplicons on the different regions of the *CYP707A1* and *CYP707A2* promoter. Schematic drawing of the *CYP707A1* (at the top of **A**) and *CYP707A2* (at the top pf **B)** locus and locations of the ChIP assay amplicons (A1-1 to A1-3, A2-1 and A2-2). *TUBULIN4* expression was assessed as the internal control. The ChIP results were presented as fold enrichment of nontarget DNA. Bars represent mean ± SD of four biological replicates with three technical replicates each. Asterisks represent significant differences from the WT (**, p-value < 0.01; Student's *t*-test).

ABI4 directly binds to the *CACCG* motif to activate the transcription of target genes, and to the *CCAC* element to repress the target genes (Finkelstein and Rock, 2002; Bossi et al., 2009). ABI4 represses the expression of *CYP707A1* and *CYP707A2* by directly binding to the *CCAC* elements in their promoter regions (Shu et al., 2013). To examine whether HDA9 binds to the promoter regions of *CYP707A1* and *CYP707A2*, we performed chromatin immunoprecipitation (ChIP) assays using fragments A1-1 to A1-3 and A2-1 to A2-2 of the *CYP707A1* and the *CYP707A2* promoter, respectively, both of which contained several CCAC motifs (Shu et al., 2013; **Figure 6**). ChIP experiments were conducted using the *hda9-1*/HDA9 (*HDA9 promoter:HDA9-HA* in the *hda9-1* mutant) complementation plant with an anti-HA antibody under drought stress conditions. ChIP-qPCR using the chromatin-immunoprecipitated DNA showed that three amplicons of the *CYP707A1* promoter under drought stress conditions were more strongly enriched than under normal conditions (**Figure 6A**). However, in the *CYP707A2* promoter, only the A2-1 amplicon was strongly enriched during drought stress (**Figure 6B**). We found that HDA9 associates with the *CCAC* motifs in the promoters of *CYP707A1* and *CYP707A2*. These results indicated that HDA9, together with ABI4, played an important role in ABA catabolism-mediated drought stress response by negatively regulating transcription of *CYP707A1* and *CYP707A2*.

### Both HDA9 and ABI4 Act in ABA-Dependent Drought Stress Response

**Figure 7:**
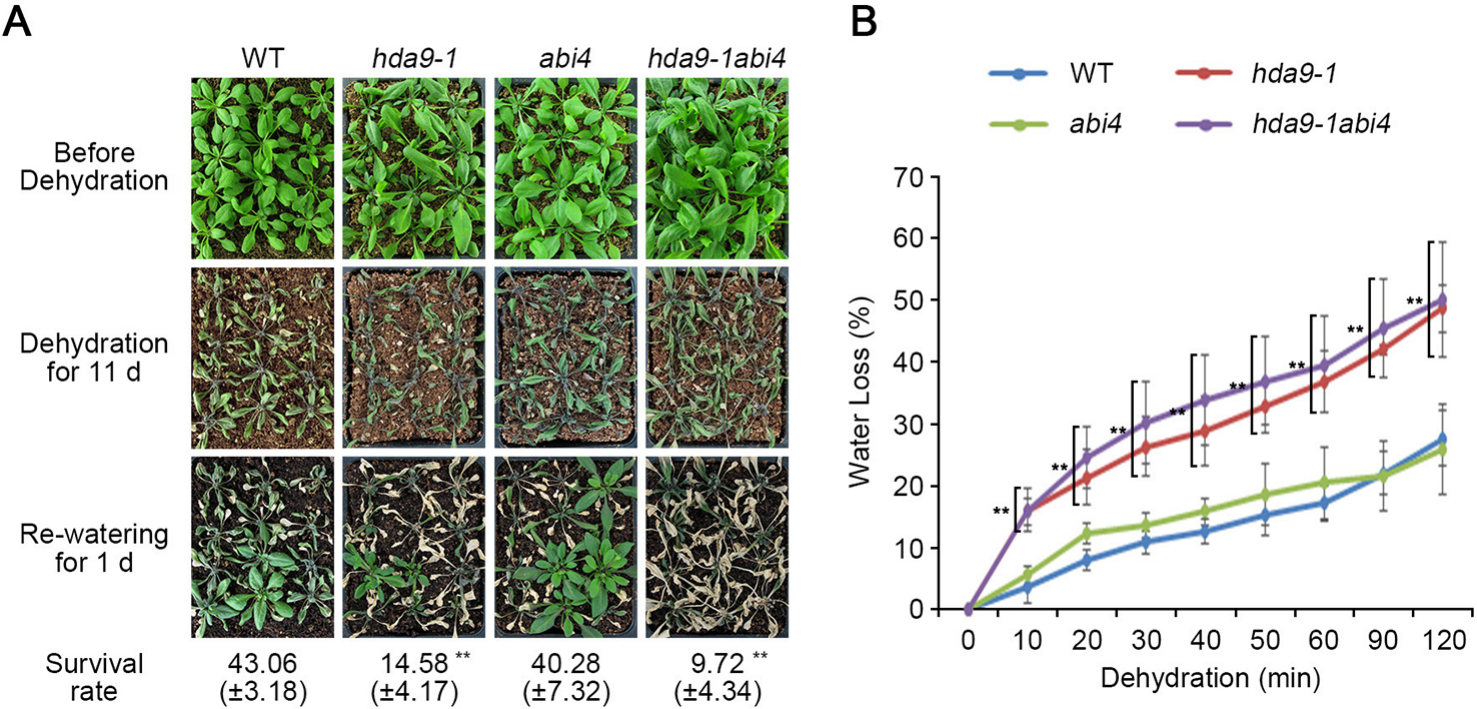
The *hda9-1abi4* double mutants are sensitive to dehydration. **(A)** One-week-old plants were transferred to soil and grown for an additional one week. Photographs of plants were taken before and after dehydration treatment. Water was withheld from two-week-old plants for 13 d, and then plants were rewatered for 1 d before the photograph was taken. Quantitation of the survival rate of WT, *hda9-1*, *abi4*, and *hda9-1abi4* plants. The values of survival rate indicated means ± SE of n = 3 biological replicates of at least 36 plants for each experiment. **(B)** Water loss assay. Water loss is presented as the percentage of weight loss versus initial fresh weight from three-week-old WT, *hda9-1*, *abi4*, and *hda9-1abi4* plants. Water loss was calculated from the results of three independent experiments. The values of survival rate indicated means ± SE of n = 3 biological replicates of at least eight plants for each experiment. Asterisks represent significant differences from the WT (**, *p*-value < 0.01; Student's *t*-test).

We found that ABA-dependent signal transduction regulated by the HDA9-ABI4 complex is essential for the ABA-mediated plant responses to drought stress. To confirm the genetic interaction between HDA9 and ABI4, we generated an *hda9-1abi4* double mutant by crossing an *hda9-1* and an *abi4* single mutant. To investigate the hypersensitivity of the *hda9-1abi4* double mutant to drought stress, we tested the phenotypes of WT, *hda9-1*, *abi4* single mutants, and the *hda9-1abi4* double mutant under drought stress conditions. When exposed to drought stress, the survival rates of the *hda9-1* and *hda9-1abi4* mutants were approximately 14.58 and 9.72%, respectively, while that of WT was approximately 43.06% (**Figure 7A**). However, drought sensitivity of *abi4* single mutant was similar with that of WT (**Figure 7**). In response to drought stress, leaf chlorosis in the *hda9-1abi4* double mutant appeared faster than in the *hda9-1* mutant (**Figure 7A**). In addition, water loss in the *hda9-1* (approximately 12.39 to 20.87%) and *hda9-1abi4* (approximately 12.21 to 23.31%) mutants was faster than in the WT and *abi4* mutant (**Figure 7B**). Water loss in the *hda9-1abi4* double mutant increased by approximately 1.51 to 4.98% than that of *hda9-1* single mutant (**Figure 7B**). Moreover, the *hda9-1* and *hda9-1abi4* mutants had more significant effects on drought-induced leaf senescence than the *abi4* mutant (**Figure 7A** and **Supplemental Figure S6**). These results suggested that HDA9 and ABI4 function together to mediate plant tolerance to drought stress.

**Figure 8:**
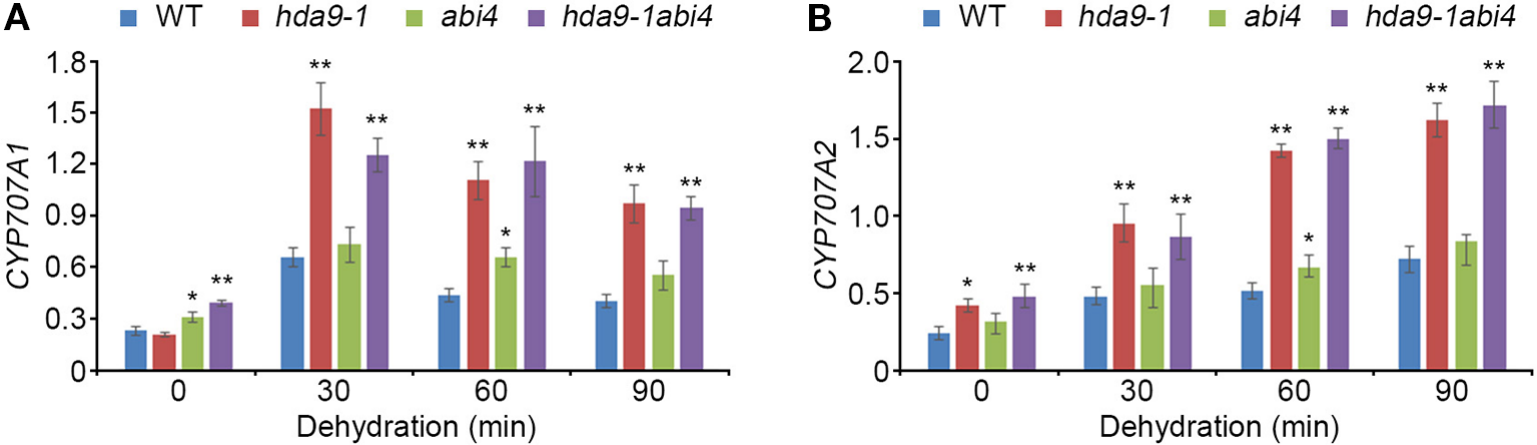
HDA9 and ABI4 regulate *CYP707A1* and *CYP707A2* expression to dehydration. **(A, B)** Quantitative RT-PCR analyses of *CYP707A1* **(A)** and *CYP707A2* **(B)** in WT, *hda9-1*, *abi4*, and *hda9-1abi4* after dehydration stress treatment. Total RNA was extracted from 10-d-old seedlings treated with dehydration stress for indicated times. Expression of *TUBULIN8* was used for normalization. Bars represent mean ± SD of three biological replicates with three technical replicates each. Asterisks represent significant differences from the WT (*, 0.01 < *p*-value ≤ 0.05; **, *p*-value < 0.01; Student's *t*-test).

To investigate the transcripts patterns of *CYP707A1* and *CYP707A2* in the *hda9-1abi4* double mutant under drought stress conditions, we performed qRT-PCR assays in WT, *hda9-1*, *abi4* single mutants, and the *hda9-1abi4* double mutant. The transcription of *CYP707A1* and *CYP707A2* was significantly up-regulated in *hda9-1* and *hda9-1abi4* double mutants compared to that in the WT (**Figure 8**). Their expression was also induced in *abi4* mutant even though the induction level is lower than that in *hda9-1* and *hda9-1abi4* double mutants. However, the transcript levels of *Lhcb1.2*, *AOX1a*, *ACS4*, and *ACS8* were not affected in the *hda9-1abi4* plants by drought stress (**Supplemental Figure S7**). These results demonstrated that HDA9 act as a key component in the ABA-dependent drought stress response.

## Discussion

**Figure 9:**
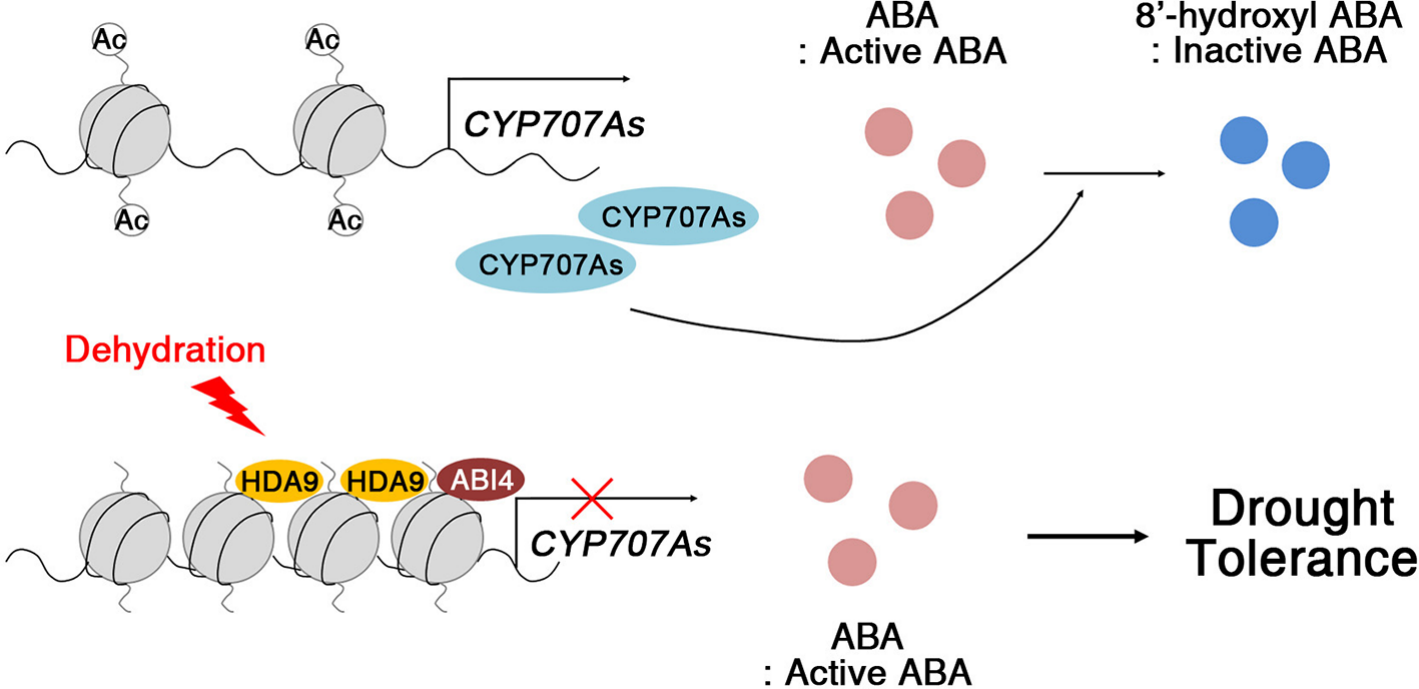
Proposed working model of HDA9 function in drought stress response. In the absence of dehydration stress, *CYP707s* are expressed and regulate ABA hydroxylation. Drought stress triggers HDA9 and ABI4 binding to promoters of *CYP707A1* and *CYP707A2* to repress expression of *CYP707s*. ABA accumulates in the plant and activates dehydration tolerance.

Members of the RPD3/HDA1 family of HDACs act as crucial components for negative regulation of gene expression in diverse developmental processes and environmental stress signaling (Tian and Chen, 2001; Tian et al., 2003; Zhou et al., 2005; Long et al., 2006; Chen and Wu, 2010). *Arabidopsis* HDA6 and HDA19 are well known for their roles in abiotic stress signaling *via* the formation of repressive complexes. HDA6 associates with HD2C and regulates the expression of abiotic stress-responsive genes, including *ABI1*, *ABI2*, and *ERF4*, through histone modifications (Luo et al., 2012b). The repressive complexes of HDA19 with ERF3, ERF4, ERF7, SIN3, and SAP18, are core chromatin remodeling complexes in abiotic stress responses, acting by mediating histone deacetylation (Song et al., 2005; Song and Galbraith, 2006). Compared with HDA6 and HDA19, less was known about how HDA9 acts in signal transduction during abiotic stress responses. Based on our results, we propose a model for the mechanism by which HDA9 modulates ABA-dependent drought stress signaling in plants (**Figure 9**). In WT plants, the expression of ABA catabolism-related genes (*CYP707As*; *e.g.*, *CYP707A1* and *CYP707A2*), changed ABA from an active to an inactive form, 8′-hydroxyl ABA, to regulate ABA homeostasis during seed germination and plant growth. However, in plants exposed to drought stress, HDA9 together with ABI4, directly represses the expression of *CYP707As*, which improved drought tolerance through the maintenance of ABA levels in the plant. Moreover, ABA levels by ABA catabolism-related genes are enough to efficiently activate drought stress-responsive gene expression, although ABA levels rapidly increased through ABA biosynthesis under drought stress. Therefore, the HDA9–ABI4 complex most likely affects a subset of the early stages of ABA-dependent signal transduction in drought stress tolerance.

### Physiological Action of HDA9 in ABA-Dependent Drought Stress Responses

Histone modifications, such as histone acetylation and deacetylation, play crucial roles in a wide range of developmental processes in plants by regulating gene expression (He et al., 2003). In particular, class I members of the RPD3/HDA1 superfamily include well-characterized HDACs, such as HDA6 and HDA19 (Hollender and Liu, 2008; Liu et al., 2014). Recent studies reported that *hda9* mutants exhibited various developmental abnormalities, including early flowering, small seedlings, slightly bulged silique tips, and reduced seed dormancy (Kim et al., 2013; van Zanten et al., 2014; Kang et al., 2015; Kim et al., 2016). The *hda9* mutant showed significant insensitivity to ABA in seed germination (**Figures 1A, B**). Consistently, it was previously reported that the seed germination of the *hda9-1* mutant was slightly enhanced compared to that of WT under 0.1 µM ABA conditions (van Zanten et al., 2014). We found that HDA9 regulated stomatal closure in the presence of exogenous ABA (**Figures 1C, D**). Moreover, the *hda9* mutants were hypersensitive to drought stress (**Figure 3**). These facts suggested that HDA9 plays an indispensable role in drought stress signal transduction by the histone modifications that determine the rate and sensitivity of downstream processes.

Drought stress promoted premature leaf senescence by phytohormone regulatory factors, particularly ABA homeostasis mechanisms (Asad et al., 2019). ABA acts as an important regulator in age-dependent physiological processes from seed germination to leaf senescence (Nambara and Marion-Poll, 2005; Wang et al., 2016; Liao et al., 2018). ABA homeostasis mechanism in ABA-mediated leaf senescence initiates from ABA biosynthesis and catabolism, ABA transport, and ABA signaling receptors (Asad et al., 2019). Then, the mechanisms of ABA-dependent leaf senescence occurred following chloroplast degradation, decline of photosynthesis, reactive oxygen species (ROS) generation, and accumulation of secondary messenger Ca^2+^ (Asad et al., 2019). We showed that leaf senescence in the *hda9-1* and *hda9-1abi4* mutants quickly progressed due to chloroplast degradation and reduced photosynthesis during drought stress (**Figure 7A** and **Supplemental Figure S6**). This suggests that HDA9 may participate in modulating leaf senescence in response to drought stress.

### HDA9–ABI4 Complex Regulates Transcriptional Cascades in ABA-Dependent Signal Transduction

ABA responses in plants were mediated by numerous diverse transcription factors, indicating that transcriptional cascades are essential in ABA signal transduction and likely involved formation of a complex with ABA-dependent *cis*-regulatory element, such as ABA response element (ABRE) and ABRE-coupling element (ABRE-CE) (Busk and Pagès, 1998; Mitsuda and Ohme-Takagi, 2009; Hubbard et al., 2010; Baldoni et al., 2015). The most common transcription factors in ABA-dependent signal transduction were ABI3, ABI4, and ABI5, which regulated ABA levels during seed dormancy and germination (Söderman et al., 2000; Nakamura et al., 2001). We showed that HDA9 physically interacts with ABI4 but does not associate with other major transcription factors in ABA responses (**Figure 3** and **Supplementary Figure S3**). ABI4 transcription factor mediates phytohormone homeostasis during seed germination by regulating ABA catabolism-related and gibberellic acid (GA) biosynthesis-related gene expressions (Shu et al., 2013). In addition, the ABI4 loss-of-function mutant (*abi4-101*) exhibits highly tolerant phenotypes to several abiotic stress, such as ABA, salt, mannitol and sugar stress (Daszkowska-Golec et al., 2013). We showed that transcriptional expression of *CYP707A1* and *CYP707A2* in the *hda9-1*, *hda9-2*, and *hda9-1abi4* mutants were significantly increased compared to that in the WT under drought stress (**Figures 4** and **8**). In addition, the ABA level in the *hda9* mutant was decreased under both non-stress and drought stress conditions (**Figure 5**). Therefore, our results demonstrated that the effect of ABA in increasing the expression of *CYP707A1* and *CYP707A2* was largely impaired in the *hda9* mutants, indicating that HDA9 is a transcriptional regulator required for the proper expression of ABA catabolism-related genes in an ABA signaling pathway.

## Conclusion

We characterized the function of HDA9, a RPD3-type HISTONE DEACETYLASE 9, in ABA-dependent drought stress response. The *hda9* mutants were tolerant during seed germination and stomata irregularity to exogenous ABA. The *hda9-1* mutants and *hda9-1abi4* double mutant were hypersensitive to drought stress, suggesting that HDA9 negatively regulates ABA catabolism-related genes, *CYP707A1* and *CYP707A2*. Our results demonstrate that HDA9 acts as an important negative regulator in transcriptional regulation of ABA catabolism-related genes, such as *CYP707A1* and *CYP707A2* in plant response to drought stress.

## Data Availability Statement

All datasets generated for this study are included in the article/**Supplementary Material**.

## Author Contributions

DB, MK, and D-JY designed the experiments. DB and GS performed most of the experiments, and MK and D-JY wrote the manuscript. SL discussed and commented on the results and manuscripts. MS performed some of the experiments. D-JY, MK, and DB provided funding for research work.

## Funding

This work was supported by the Next Generation BioGreen21 Program [SSAC, grant number PJ01318201 (to D-JY) and PJ01318202 (to MK)], the Rural Development Administration Republic of Korea, and the Basic Science Research Program through the National Research Foundation of Korea (NRF) funded by the Ministry of Education [2015R1A6A1A03031413 (to MK), 2016R1D1A1B01011803 (to DB) and Global Research Laboratory 2017K1A1A2013146 (to D-JY)].

## Conflict of Interest

The authors declare that the research was conducted in the absence of any commercial or financial relationships that could be construed as a potential conflict of interest.

## References

[B1] Asad M. A. U. Zakari S. A. Zhao Q. Zhou L. Ye Y. Cheng F. (2019). Abiotic stresses intervene with aba signaling to induce destructive metabolic pathways leading to death: premature leaf senescence in plants. Int. J. Mol. Sci. 20 (2), E256. 10.3390/ijms20020256 30634648 PMC6359161

[B2] Aufsatz W. Mette M. F. van der Winden J. Matzke M. Matzke A. J. (2002). HDA6, a putative histone deacetylase needed to enhance DNA methylation induced by double-stranded RNA. EMBO J. 21 (24), 6832–6841. 10.1093/emboj/cdf663 12486004 PMC139084

[B3] Baldoni E. Genga A. Cominelli E. (2015). Plant MYB transcription factors: their role in drought response mechanisms. Int. J. Mol. Sci. 16 (7), 15811–15851. 10.3390/ijms160715811 26184177 PMC4519927

[B4] Blatt M. R. (2000). Cellular signaling and volume control in stomatal movements in plants. Ann. Rev. Cell Dev. Biol. 16, 221–241. 10.1146/annurev.cellbio.16.1.221 11031236

[B5] Bohnert H. J. Sheveleva E. (1998). Plant stress adaptations-making metabolism move. Curr. Opin. Plant Biol. 1 (3), 267–274. 10.1016/S1369-5266(98)80115-5 10066591

[B6] Bossi F. Cordoba E. Dupré P. Mendoza M. S. Román C. S. León P. (2009). The Arabidopsis ABA-INSENSITIVE (ABI) 4 factor acts as a central transcription activator of the expression of its own gene, and for the induction of ABI5 and SBE2.2 genes during sugar signaling. Plant J. 59 (3), 359–374. 19392689 10.1111/j.1365-313X.2009.03877.x

[B7] Busk P. K. Pagès M. (1998). Regulation of abscisic acid-induced transcription. Plant Mol. Biol. 37 (3), 425–435. 10.1023/A:1006058700720 9617810

[B8] Chen L. T. Wu K. (2010). Role of histone deacetylases HDA6 and HDA19 in ABA and abiotic stress response. Plant Signal Behav. 5 (10), 1318–1320. 10.4161/psb.5.10.13168 20930557 PMC3115378

[B9] Chen X. Lu L. Mayer K. S. Scalf M. Qian S. Lomax A. (2016). POWERDRESS interacts with histone deacetylase 9 to promote aging in arabidopsis. Elife 5, e17214. 10.7554/eLife.17214 27873573 PMC5119886

[B10] Cigliano R. A. Cremona G. Paparo R. Termolino P. Perrella G. Gutzat R. (2013). Histone deacetylase AtHDA7 is required for female gametophyte and embryo development in Arabidopsis. Plant Physiol. 163 (1), 431–440. 10.1104/pp.113.221713 23878078 PMC3762662

[B11] Cutler S. R. Rodriguez P. L. Finkelstein R. R. Abrams S. R. (2010). Abscisic acid: emergence of a core signaling network. Annu. Rev. Plant Biol. 61, 651–679. 10.1146/annurev-arplant-042809-112122 20192755

[B12] Daszkowska-Golec A. Wojnar W. Rosikiewicz M. Szarejko I. Maluszynski M. Szweykowska-Kulinska Z. (2013). Arabidopsis suppressor mutant of abh1 shows a new face of the already known players: ABH1 (CBP80) and ABI4-in response to ABA and abiotic stresses during seed germination. Plant Mol. Biol. 81 (1-2), 189–209. 23196831 10.1007/s11103-012-9991-1 PMC3527740

[B13] Dong T. Park Y. Hwang I. (2015). Abscisic acid: biosynthesis, inactivation, homoeostasis and signalling. Essays Biochem. 58, 29–48. 10.1042/bse0580029 26374885

[B14] Dong Z. Yu Y. Li S. Wang J. Tang S. Huang R. (2016). Abscisic acid antagonizes ethylene production through the ABI4-mediated transcriptional repression of ACS4 and ACS8 in Arabidopsis. Mol. Plant 9 (1), 126–135. 10.1016/j.molp.2015.09.007 26410794

[B15] Finkelstein R. R. Lynch T. J. (2000). Abscisic acid inhibition of radicle emergence but not seedling growth is suppressed by sugars. Plant Physiol. 122 (4), 1179–1186. 10.1104/pp.122.4.1179 10759513 PMC58952

[B16] Finkelstein R. R. Rock C. D. (2002). Abscisic acid biosynthesis and response. Arabidopsis Book. 1, e0058. 10.1199/tab.0058 22303212 PMC3243367

[B17] Finkelstein R. R. Gampala S. S. Rock C. D. (2002). Abscisic acid signaling in seeds and seedlings. Plant Cell. Suppl, S15–S45. 10.1105/tpc.010441 PMC151246 12045268

[B18] Finkelstein R. Reeves W. Ariizumi T. Steber C. (2008). Molecular aspects of seed dormancy. Annu. Rev. Plant Biol. 59, 387–415. 10.1146/annurev.arplant.59.032607.092740 18257711

[B19] Giraud E. Van Aken O. Ho L. H. Whelan J. (2009). The transcription factor abi4 is a regulator of mitochondrial retrograde expression of alternative oxidase1a. Plant Physiol. 150 (3), 1286–1296. 10.1104/pp.109.139782 19482916 PMC2705018

[B20] He Y. Michaels S. D. Amasino R. M. (2003). Regulation of flowering time by histone acetylation in Arabidopsis. Science 302 (5651), 1751–1754. 10.1126/science.1091109 14593187

[B21] Hollender C. Liu Z. (2008). Histone deacetylase genes in Arabidopsis development. J. Integr. Plant Biol. 50 (7), 875–885. 10.1111/j.1744-7909.2008.00704.x 18713398

[B22] Hubbard K. E. Nishimura N. Hitomi K. Getzoff E. D. Schroeder J. I. (2010). Early abscisic acid signal transduction mechanisms: newly discovered components and newly emerging questions. Genes Dev. 24 (16), 1695–1708. 10.1101/gad.1953910 20713515 PMC2922499

[B23] Jacobsen J. V. Pearce D. W. Poole A. T. Pharis R. P. Mander L. N. (2002). Abscisic acid, phaseic acid and gibberellin contents associated with dormancy and germination in barley. Physiol. Plant 115 (3), 428–441. 10.1034/j.1399-3054.2002.1150313.x 12081536

[B24] König A. C. Hartl M. Pham P. A. Laxa M. Boersema P. J. Orwat A. (2014). The Arabidopsis class II sirtuin is a lysine deacetylase and interacts with mitochondrial energy metabolism. Plant Physiol. 164 (3), 1401–1414. 10.1104/pp.113.232496 24424322 PMC3938629

[B25] Kang M. J. Jin H. S. Noh Y. S. Noh B. (2015). Repression of flowering under a noninductive photoperiod by the HDA9- AGL19-FT module in Arabidopsis. New Phytol. 206 (1), 281–294. 10.1111/nph.13161 25406502

[B26] Kim W. Latrasse D. Servet C. Zhou D. X. (2013). Arabidopsis histone deacetylase HDA9 regulates flowering time through repression of AGL19. Biochem. Biophys. Res. Commun. 432 (2), 394–398. 10.1016/j.bbrc.2012.11.102 23237803

[B27] Kim Y. J. Wang R. Gao L. Li D. Xu C. Mang H. (2016). POWERDRESS and HDA9 interact and promote histone H3 deacetylation at specific genomic sites in Arabidopsis. Proc. Natl. Acad. Sci. U.S.A. 113 (51), 14858–14863. 10.1073/pnas.1618618114 27930340 PMC5187680

[B28] Koussevitzky S. Nott A. Mockler T. C. Hong F. Sachetto-Martins G. Surpin M. (2007). Signals from chloroplasts converge to regulate nuclear gene expression. Science 316 (5825), 715–719. 10.1126/science.1140516 17395793

[B29] Kushiro T. Okamoto M. Nakabayashi K. Yamagishi K. Kitamura S. Asami T. (2004). The Arabidopsis cytochrome P450 CYP707A encodes ABA 8'-hydroxylases: key enzymes in ABA catabolism. EMBO J. 23 (7), 1647–1656. 10.1038/sj.emboj.7600121 15044947 PMC391058

[B30] Lee K. H. Piao H. L. Kim H. Y. Choi S. M. Jiang F. Hartung W. (2006). Activation of glucosidase *via* stress-induced polymerization rapidly increases active pools of abscisic acid. Cell 126 (6), 1109–1120. 10.1016/j.cell.2006.07.034 16990135

[B31] Lee K. Park O. S. Jung S. J. Seo P. J. (2016). Histone deacetylation-mediated cellular dedifferentiation in Arabidopsis. J. Plant Physiol. 191, 95–100. 10.1016/j.jplph.2015.12.006 26724747

[B32] Liao Y. Bai Q. Xu P. Wu T. Guo D. Peng Y. (2018). Mutation in rice abscisic Acid2 results in cell death, enhanced disease-resistance, altered seed dormancy and development. Front. Plant Sci. 9, 405. 10.3389/fpls.2018.00405 29643863 PMC5882781

[B33] Liu C. Li L. C. Chen W. Chen X. Xu Z. H. Bai S. N. (2013). HDA18 affects cell fate in Arabidopsis root epidermis *via* histone acetylation at four kinase genes. Plant Cell. 25 (1), 257–269. 10.1105/tpc.112.107045 23362208 PMC3584540

[B34] Liu X. Yang S. Zhao M. Luo M. Yu C. W. Chen C. Y. (2014). Transcriptional repression by histone deacetylases in plants. Mol. Plant 7 (5), 764–772. 10.1093/mp/ssu033 24658416

[B35] Long J. A. Ohno C. Smith Z. R. Meyerowitz E. M. (2006). TOPLESS regulates apical embryonic fate in Arabidopsis. Science 312 (5779), 1520–1523. 10.1126/science.1123841 16763149

[B36] Lopez-Molina L. Mongrand S. Chua N. H. (2001). A postgermination developmental arrest checkpoint is mediated by abscisic acid and requires the ABI5 transcription factor in Arabidopsis. Proc. Natl. Acad. Sci. U. S. A. 98 (8), 4782–4787. 10.1073/pnas.081594298 11287670 PMC31911

[B37] Lopez-Molina L. Mongrand S. McLachlin D. Chait B. Chua N. H. (2002). ABI5 acts downstream of ABI3 to execute an ABA-dependent growth arrest during germination. Plant J. 32 (3), 317–328. 10.1046/j.1365-313X.2002.01430.x 12410810

[B38] Luo M. Liu X. Singh P. Cui Y. Zimmerli L. Wu K. (2012a). Chromatin modifications and remodeling in plant abiotic stress responses. Biochim. Biophys. Acta 1819 (2), 129–136. 10.1016/j.bbagrm.2011.06.008 21708299

[B39] Luo M. Wang Y. Y. Liu X. Yang S. Lu Q. Cui Y. (2012b). HD2C interacts with HDA6 and is involved in ABA and salt stress response in Arabidopsis. J. Exp. Bot. 63 (8), 3297–3306. 10.1093/jxb/ers059 22368268 PMC3350937

[B40] Mitsuda N. Ohme-Takagi M. (2009). Functional analysis of transcription factors in Arabidopsis. Plant Cell Physiol. 50 (7), 1232–1248. 10.1093/pcp/pcp075 19478073 PMC2709548

[B41] Nakamura S. Lynch T. J. Finkelstein R. R. (2001). Physical interactions between ABA response loci of Arabidopsis. Plant J. 26 (6), 627–635. 10.1046/j.1365-313x.2001.01069.x 11489176

[B42] Nambara E. Marion-Poll A. (2005). Abscisic acid biosynthesis and catabolism. Annu. Rev. Plant Biol. 56, 165–185. 10.1146/annurev.arplant.56.032604.144046 15862093

[B43] Okamoto M. Kuwahara A. Seo M. Kushiro T. Asami T. Hirai N. (2006). CYP707A1 and CYP707A2, which encode abscisic acid 8'-hydroxylases, are indispensable for proper control of seed dormancy and germination in Arabidopsis. Plant Physiol. 141 (1), 97–107. 10.1104/pp.106.079475 16543410 PMC1459320

[B44] Pan Y. Michael T. P. Hudson M. E. Kay S. A. Chory J. Schuler M. A. (2009). Cytochrome P450 monooxygenases as reporters for circadian-regulated pathways. Plant Physiol. 150 (2), 858–878. 10.1104/pp.108.130757 19386812 PMC2689971

[B45] Pandey R. Müller A. Napoli C. A. Selinger D. A. Pikaard C. S. Richards E. J. (2002). Analysis of histone acetyltransferase and histone deacetylase families of Arabidopsis thaliana suggests functional diversification of chromatin modification among multicellular eukaryotes. Nucleic Acids Res. 30 (23), 5036–5055. 10.1093/nar/gkf660 12466527 PMC137973

[B46] Park J. Lim C. J. Khan I. U. Jan M. Khan H. A. Park H. J. (2018). Identification and molecular characterization of HOS15-interacting proteins in Arabidopsis thaliana. J. Plant Biol. 61, 336–345. 10.1007/s12374-018-0313-2

[B47] Penfield S. Li Y. Gilday A. D. Graham S. Graham I. A. (2006). Arabidopsis ABA INSENSITIVE4 regulates lipid mobilization in the embryo and reveals repression of seed germination by the endosperm. Plant Cell. 18 (8), 1887–1899. 10.1105/tpc.106.041277 16844907 PMC1533976

[B48] Raghavendra A. S. Gonugunta V. K. Christmann A. Grill E. (2010). ABA perception and signalling. Trends Plant Sci. 15 (7), 395–401. 10.1016/j.tplants.2010.04.006 20493758

[B49] Ryu H. Cho H. Bae W. Hwang I. (2014). Control of early seedling development by BES1/TPL/HDA19-mediated epigenetic regulation of ABI3. Nat. Commun. 5, 4138. 10.1038/ncomms5138 24938150

[B50] Söderman E. M. Brocard I. M. Lynch T. J. Finkelstein R. R. (2000). Regulation and function of the Arabidopsis ABA-insensitive4 gene in seed and abscisic acid response signaling networks. Plant Physiol. 124 (4), 1752–1765. 10.1104/pp.124.4.1752 11115891 PMC59872

[B51] Saleh A. Alvarez-Venegas R. Avramova Z. (2008). An efficient chromatin immunoprecipitation (ChIP) protocol for studying histone modifications in Arabidopsis plants. Nat. Protoc. 3 (6), 1018–1025. 10.1038/nprot.2008.66 18536649

[B52] Shu K. Zhang H. Wang S. Chen M. Wu Y. Tang S. (2013). ABI4 regulates primary seed dormancy by regulating the biogenesis of abscisic acid and gibberellins in Arabidopsis. PloS Genet. 9 (6), e1003577. 10.1371/journal.pgen.1003577 23818868 PMC3688486

[B53] Shu K. Liu X. D. Xie Q. He Z. H. (2016). Two faces of one seed: hormonal regulation of dormancy and germination. Mol. Plant 9 (1), 34–45. 10.1016/j.molp.2015.08.010 26343970

[B54] Song C. P. Galbraith D. W. (2006). AtSAP18, an orthologue of human SAP18, is involved in the regulation of salt stress and mediates transcriptional repression in Arabidopsis. Plant Mol. Biol. 60 (2), 241–257. 10.1007/s11103-005-3880-9 16429262

[B55] Song C. P. Agarwal M. Ohta M. Guo Y. Halfter U. Wang P. (2005). Role of an Arabidopsis AP2/EREBP-type transcriptional repressor in abscisic acid and drought stress responses. Plant Cell. 17 (8), 2384–2396. 10.1105/tpc.105.033043 15994908 PMC1182496

[B56] Sridha S. Wu K. (2006). Identification of AtHD2C as a novel regulator of abscisic acid responses in Arabidopsis. Plant J. 46 (1), 124–133. 10.1111/j.1365-313X.2006.02678.x 16553900

[B57] Stone S. L. Williams L. A. Farmer L. M. Vierstra R. D. Callis J. (2006). Keep on going, a ring e3 ligase essential for arabidopsis growth and development, is involved in abscisic acid signaling. Plant Cell 18 (12), 3415–3428. 10.1105/tpc.106.046532 17194765 PMC1785414

[B58] Tian L. Chen Z. J. (2001). Blocking histone deacetylation in Arabidopsis induces pleiotropic effects on plant gene regulation and development. Proc. Natl. Acad. Sci. U.S.A. 98 (1), 200–205. 10.1073/pnas.98.1.200 11134508 PMC14568

[B59] Tian L. Wang J. Fong M. P. Chen M. Cao H. Gelvin S. B. (2003). Genetic control of developmental changes induced by disruption of Arabidopsis histone deacetylase 1 (AtHD1) expression. Genetics 165 (1), 399–409. 14504245 10.1093/genetics/165.1.399 PMC1462737

[B60] van Zanten M. Zöll C. Wang Z. Philipp C. Carles A. Li Y. (2014). Histone deacetylase 9 represses seedling traits in Arabidopsis thaliana dry seeds. Plant J. 80 (3), 475–488. 10.1111/tpj.12646 25146719

[B61] Verdin E. Ott M. (2015). 50 years of protein acetylation: from gene regulation to epigenetics, metabolism and beyond. Nat. Rev. Mol. Cell Biol. 16 (4), 258–264. 10.1038/nrm3931 25549891

[B62] Vishwakarma K. Upadhyay N. Kumar N. Yadav G. Singh J. Mishra R. K. (2017). Abscisic acid signaling and abiotic stress tolerance in plants: a review on current knowledge and future prospects. Front. Plant Sci. 8, 161. 10.3389/fpls.2017.00161 28265276 PMC5316533

[B63] Wang Z. Cao H. Chen F. Liu Y. (2014). The roles of histone acetylation in seed performance and plant development. Plant Physiol. Biochem. 84, 125–133. 10.1016/j.plaphy.2014.09.010 25270163

[B64] Wang F. Liu J. Chen M. Zhou L. Li Z. Zhao Q. (2016). Involvement of abscisic acid in psii photodamage and d1 protein turnover for light-induced premature senescence of rice flag leaves. PloS One 11 (8), e0161203. 10.1371/journal.pone.0161203 27532299 PMC4988704

[B65] Xiong L. Zhu J. K. (2003). Regulation of abscisic acid biosynthesis. Plant Physiol. 133 (1), 29–36. 10.1104/pp.103.025395 12970472 PMC523868

[B66] Xu W. Jia L. Shi W. Liang J. Zhou F. Li Q. (2013). Abscisic acid accumulation modulates auxin transport in the root tip to enhance proton secretion for maintaining root growth under moderate water stress. New Phytol. 197 (1), 139–150. 10.1111/nph.12004 23106247

[B67] Yamaguchi-Shinozaki K. Shinozaki K. (2006). Transcriptional regulatory networks in cellular responses and tolerance to dehydration and cold stresses. Annu. Rev. Plant Biol. 57, 781–803. 10.1146/annurev.arplant.57.032905.105444 16669782

[B68] Yuan K. Wysocka-Diller J. (2006). Phytohormone signaling pathways interact with sugars during seed germination and seedling development. J. Exp. Bot. 57 (12), 3359–3367. 10.1093/jxb/erl096 16916886

[B69] Yuan L. Liu X. Luo M. Yang S. Wu K. (2013). Involvement of histone modifications in plant abiotic stress responses. J. Integr. Plant Biol. 55 (10), 892–901. 10.1111/jipb.12060 24034164

[B70] Zhang Z. W. Feng L. Y. Cheng J. Tang H. Xu F. Zhu F. (2013). The roles of two transcription factors, ABI4 and CBFA, in ABA and plastid signalling and stress responses. Plant Mol. Biol. 83 (4-5), 445–458. 10.1007/s11103-013-0102-8 23832569

[B71] Zheng Y. Ding Y. Sun X. Xie S. Wang D. Liu X. (2016). Histone deacetylase HDA9 negatively regulates salt and drought stress responsiveness in Arabidopsis. J. Exp. Bot. 67 (6), 1703–1713. 10.1093/jxb/erv562 26733691

[B72] Zhou C. Labbe H. Sridha S. Wang L. Tian L. Latoszek-Green M. (2004). Expression and function of HD2-type histone deacetylases in Arabidopsis development. Plant J. 38 (5), 715–724. 10.1111/j.1365-313X.2004.02083.x 15144374

[B73] Zhou C. Zhang L. Duan J. Miki B. Wu K. (2005). HISTONE DEACETYLASE19 is involved in jasmonic acid and ethylene signaling of pathogen response in Arabidopsis. Plant Cell. 17 (4), 1196–1204. 10.1105/tpc.104.028514 15749761 PMC1087996

[B74] Zhu J. K. (2001). Cell signaling under salt, water and cold stresses. Curr. Opin. Plant Biol. 4 (5), 401–406. 10.1016/S1369-5266(00)00192-8 11597497

