# Supplementary material for: Histone Deacetylase HDA9 With ABI4 Contributes to Abscisic Acid Homeostasis in Drought Stress Response
Source: Front Plant Sci. 2020 Feb 25;11:143. doi: 10.3389/fpls.2020.00143 (PMC7052305; doi:10.3389/fpls.2020.00143)
Supplement: Supplementary file 1 [file Table_1.docx]

***Supplementary Materials***

**Title: Histone deacetylase HDA9 with ABI4 contributes to ABA homeostasis in drought stress response**

Dongwon Baek^a, †^, Gilok Shin^c, †^, Min Chul Kim^a, b, †^, Mingzhe Shen^a^, Sang Yeol Lee^a^, Dae-Jin Yun^c,^ *

^a^ Division of Applied Life Science (BK21plus program), Plant Molecular Biology and Biotechnology Research Center, Gyeongsang National University, Jinju 52828, Korea

^b^ Institute of Agriculture & Life Science, Gyeongsang National University, Jinju 52828, Korea

^c^ Department of Biomedical Science and Engineering, Konkuk University, Seoul 05029, Korea

*** Corresponding author:**

Department of Biomedical Science and Engineering, Konkuk University, Seoul 05029, Korea Phone: +82 2 450 0583

E-Mail: Dae-Jin Yun ([djyun@konkuk.ac.kr](mailto:djyun@konkuk.ac.kr))

^†^ These authors contributed equally to this work.

**1. Supplementary Figures and Tables**

**1. 1. Supplementary Figures**

**
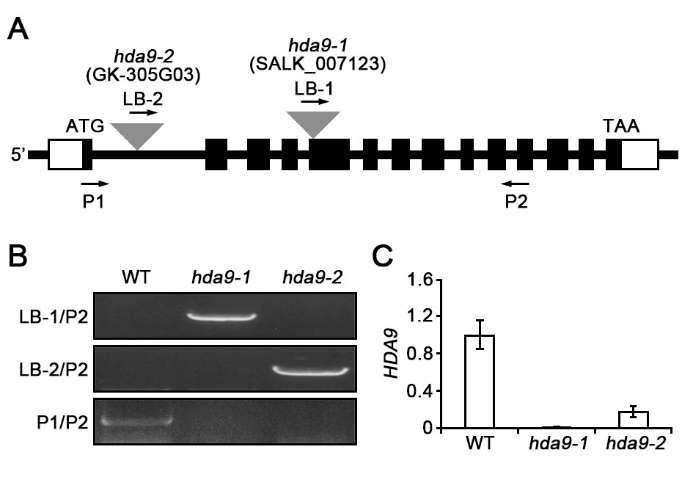
**

**Supplementary Figure S1.** Identification of *hda9* mutants.

(A) Schematic structure of the *hda9* allele with the T-DNA insertion. Introns are depicted as line, and exons are represented as black boxes. Inverted triangles indicate the location of the T-DNA insertion site for *hda9-1* and *hda9-2*. (B) Genotyping PCR of *hda9-1* and *hda9-2* loss-of-function mutant plants. Primers used in genotyping PCR are listed in Table 1. (C) Quantitative PCR analysis of *HDA9* expression WT, *hda9-1*, and *hda9-2* plants. Total RNA was extracted from 10-d-old seedling plants and *HDA9* expression was detected by qRT-PCR analysis. Expression of *TUBULIN8* was used for normalization. Bars represent mean ± SD of three biological replicates with three technical replicates each. Asterisks represent significant differences from the WT (*, 0.01 < *p*-value ≤ 0.05; **, *p*-value < 0.01; Student's *t*-test).


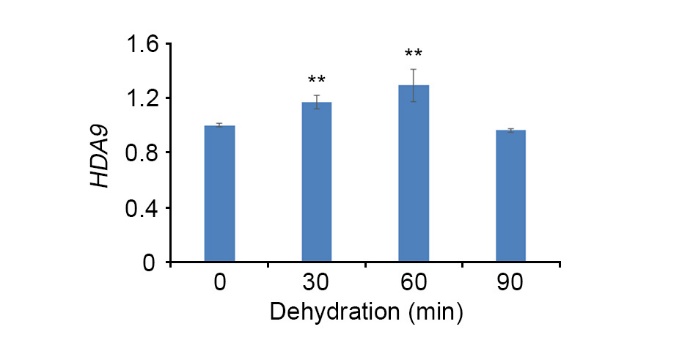


**Supplementary Figure S2.** The transcriptional expression of *HDA9* in WT in response to dehydration.

Quantitative RT-PCR analyses of *HDA9* in WT after dehydration stress treatment. Total RNA was extracted from 10-d-old WT seedlings treated with dehydration stress for indicated times. Expression of *TUBULIN8* was used for normalization. Bars represent mean ± SD of three biological replicates with three technical replicates each. Asterisks represent significant differences from the 0 time (**, p-value < 0.01; Student's t-test).


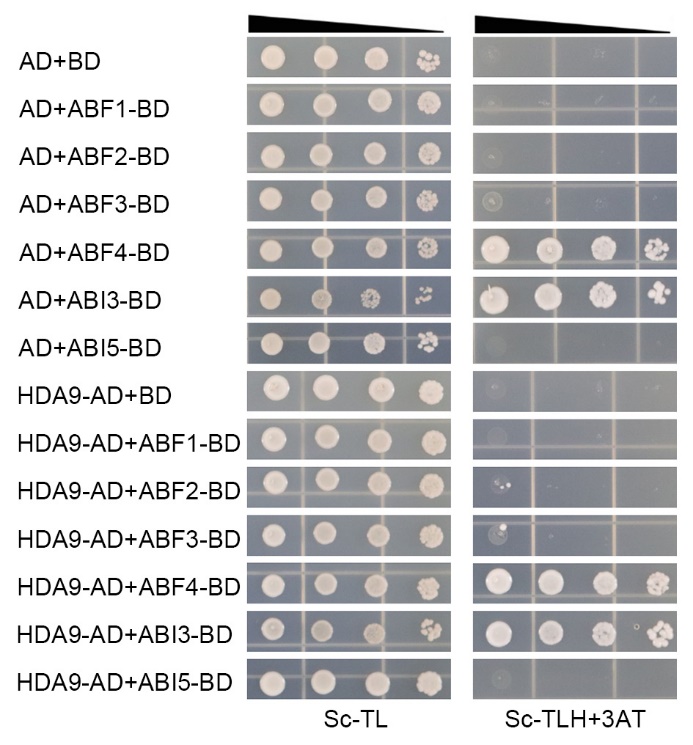


**Supplementary Figure S3.** HDA9 does not interact with other ABA-related transcription factors.

BD, *pDEST32* is the bait plasmid; AD, *pDEST22* the prey plasmid. The co-transformed yeast strains were plated on the control SD-TL and selective medium SD-TLH plus 25 mM 3-AT. The combinations with empty plasmid were used as negative controls.


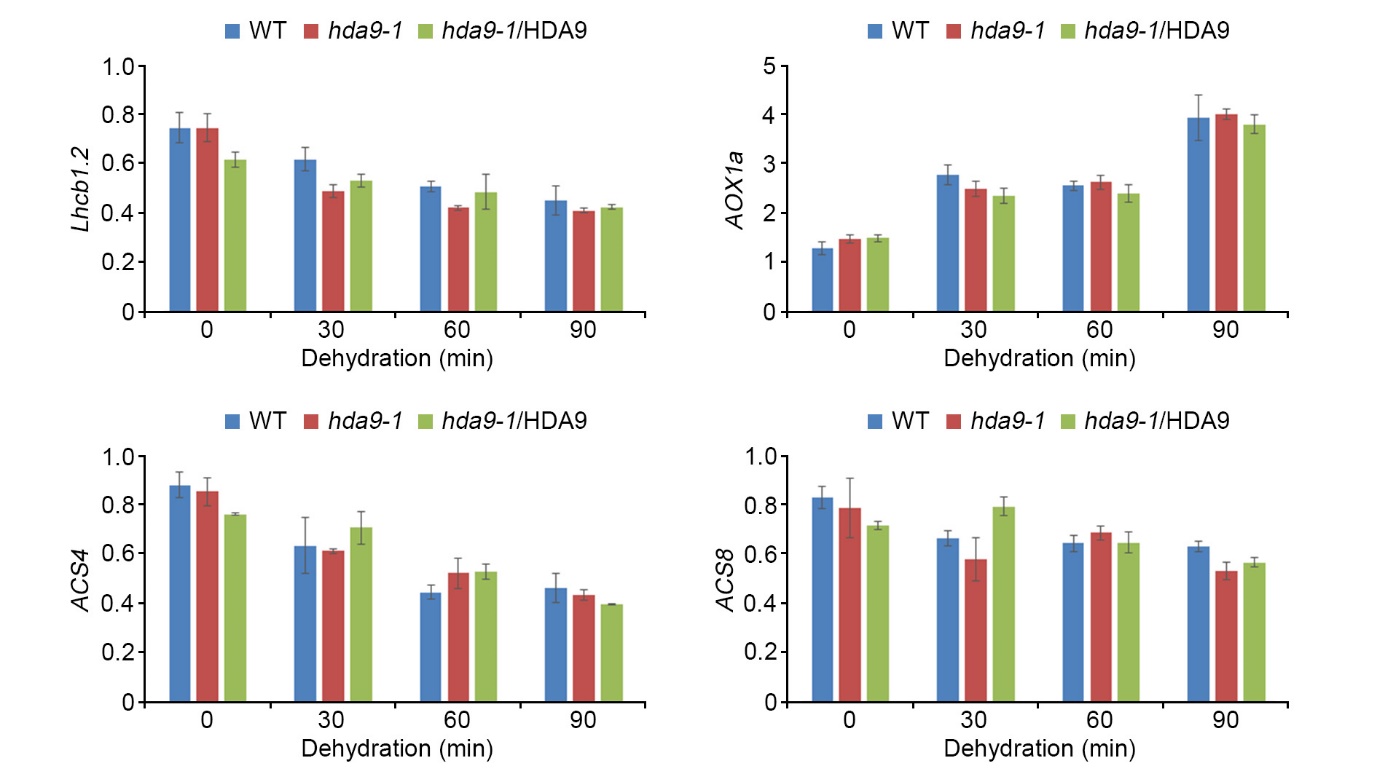


**Supplementary Figure S4.** HDA9 does not regulate expression of other ABI4-targeted genes under dehydration stress.

Quantitative RT-PCR analyses of other ABI4-targeted genes (*Lhcb1.2*, *AOX1a*, *ACS4*, and *ACS8*) in WT, *hda9-1* and *hda9-1*/HDA9 after dehydration stress treatment. Total RNA was extracted from 10-d-old seedlings treated with dehydration stress for indicated times. Expression of *TUBULIN8* was used for normalization. Bars represent mean ± SD of three biological replicates with three technical replicates each. Asterisks represent significant differences from the WT (*, 0.01 < *p*-value ≤ 0.05; **, *p*-value < 0.01; Student's *t*-test).


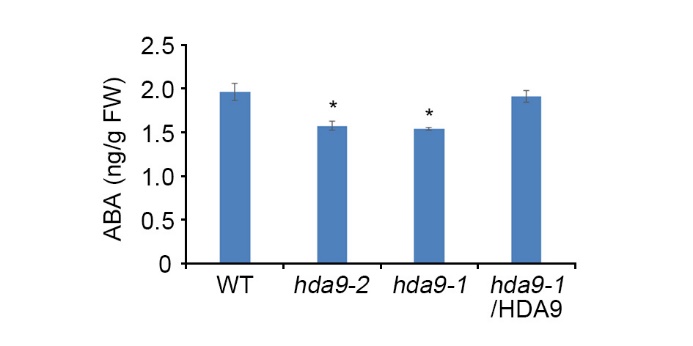


**Supplementary Figure S5.** HDA9 regulates intracellular ABA levels in dry seeds.

ABA content was measured from dry seeds of WT, *hda9-1*, *hda9-2*, and *hda9-1*/HDA9. Error bars represent the SD from three independent experiments. Asterisks represent significant differences from the WT (*, *p*-value ≤ 0.05, Student’s *t*-test).


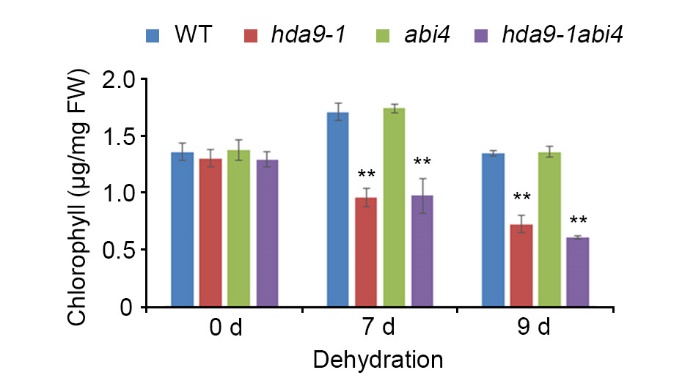


**Supplementary Figure S6.** HDA9 regulates chlorophyll contents in leaves under drought stress.

Total chlorophyll contents were measured in two-week-old WT, *hda9-1*, *abi4*, and *hda9-1abi4* after dehydration stress treatment. Total chlorophyll was extracted from leaves of each plants treated with dehydration stress for indicated times. Chlorophyll was analyzed by using UV spectrophotometer at 645 nm (chlorophyll B) and 663 nm (chlorophyll A). Total chlorophyll was calculated using fallowing formula:

Chlorophyll (µg/mg F.W.) = [ (OD_645_ x 20.2) + (OD_663_ x 8) ] / Fresh weight (mg)

Error bars represent the SD from three independent experiments. Asterisks represent significant differences from the WT (**, *p*-value < 0.01; Student's *t*-test).


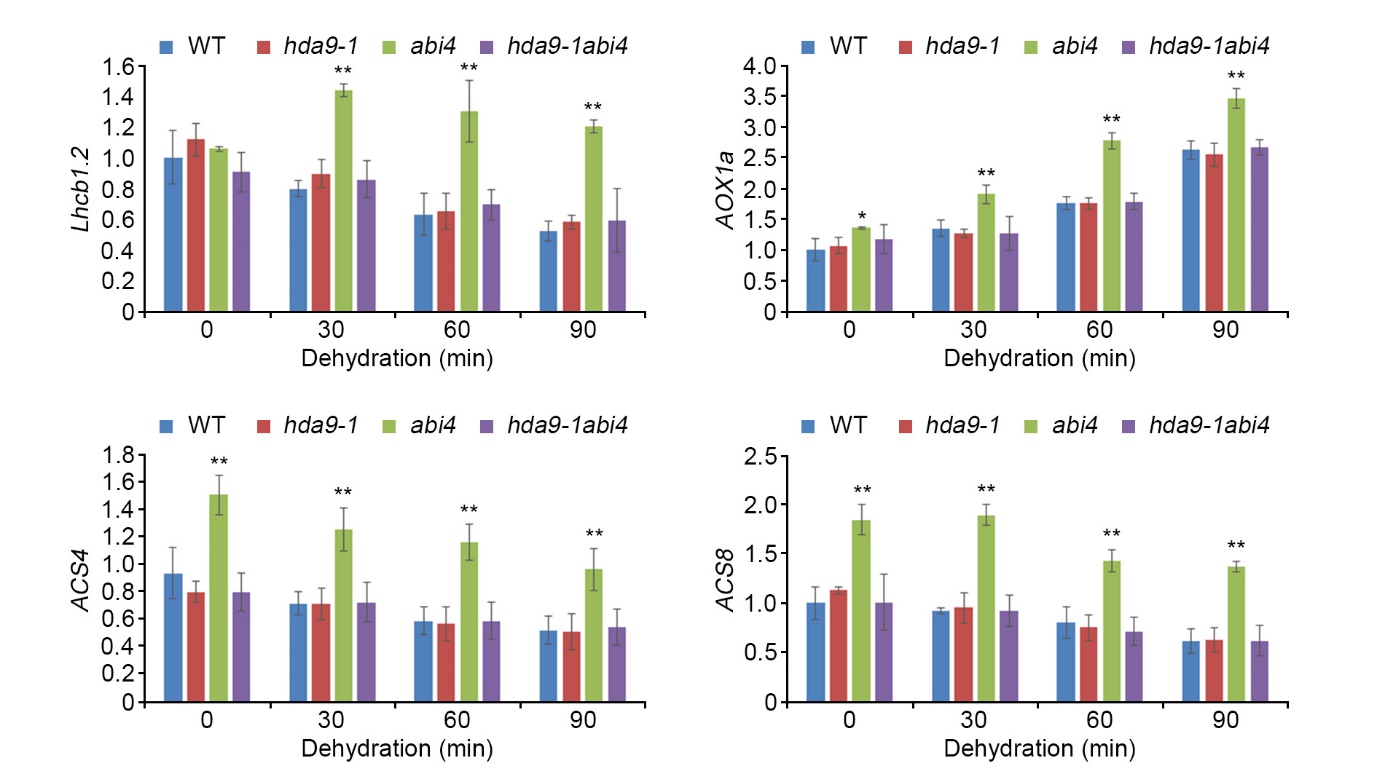


**Supplementary Figure S7.** HDA9 and ABI4 do not regulate expression of other ABI4 downstream genes under dehydration stress.

Quantitative RT-PCR analyses of other ABI4-targeted genes (*Lhcb1.2*, *AOX1a*, *ACS4*, and *ACS8*) in WT, *hda9-1*, *abi4*, and *hda9-1abi4* after dehydration stress treatment. Total RNA was extracted from 10-d-old seedlings treated with dehydration stress for indicated times. Expression of *TUBULIN8* was used for normalization. Bars represent mean ± SD of three biological replicates with three technical replicates each. Asterisks represent significant differences from the WT (*, 0.01 < *p*-value ≤ 0.05; **, *p*-value < 0.01; Student's *t*-test).

**1. 2. Supplementary Table**

**Supplementary Table S1**. Lists of putative target genes of ABI4 transcription factor

| Target Genes | Functions | Regulation | References |
| --- | --- | --- | --- |
| Lhcb1.2 | Chloroplast retrograde signal | Negative regulation | Koussevitzky et al., 2007 |
| AOX1a | Mitochondrial retrograde signal | Negative regulation | Giraud et al., 2009 |
| CYP707A1  CYP707A2 | Seed dormancy | Negative regulation | Shu et al., 2013 |
| ACS4  ACS8 | Ethylene production | Negative regulation | Dong et al., 2016 |

**Supplementary Table S2.** Primer list used in this study

| **Name** | **Sequences (5’→3’)** | **Purpose** |
| --- | --- | --- |
| hda9 P1 | ATGGCCTGCATAGCAAGATG | Mutant genotyping |
| hda9 P2 | AAAAATCCGGCGCAAAATAC |  |
| LBb1.3 | ATTTTGCCGATTTCGGAAC |  |
| attB1 adapter | GGGGACAAGTTTGTACAAAAAAGCAGGCT | Plasmid cloning |
| attB2 adapter | GGGGACCACTTTGTACAAGAAAGCTGGGT |  |
| HDA9 attB1 | AAAAAAGCAGGCTTA ATGCGTTCCAAGGACAAAATCTC |  |
| HDA9 attB2 | AGAAAGCTGGGTCTTATGACGCATCGTTATCGTTGTC |  |
| HDA9 no attB2 | AGAAAGCTGGGTCTGACGCATCGTTATCGTTGTCTCC |  |
| ABI4 attB1 | AAAAAAGCAGGCTTAATGGACCCTTTAGCTTCCCA |  |
| ABI4 attB2 | AGAAAGCTGGGTCTTAATAGAATTCCCCCA |  |
| ABI4 no attB2 | AGAAAGCTGGGTCATAGAATTCCCCCA |  |
| ABI2-attB2 | AGAAAGCTGGGTCTCAATTCAAGGATTTGCTCT |  |
| CYP707A1 A1-1 F | CCTTCACATCTCCCACTTGT | ChIP assay |
| CYP707A1 A1-1 R | GTGAGAAACAAGGCGGAGAT |  |
| CYP707A1 A1-2 F | TCCCACTCTTTTATTCACTC |  |
| CYP707A1 A1-2 R | AAATGTGGGGTAAAGTCTAC |  |
| CYP707A1 A1-3 F | AAAGATGATGAGGATTCGGT |  |
| CYP707A1 A1-3 R | GTAAAATGAAGATGTGTGGC |  |
| CYP707A2 A2-1 F | CGTGGATTTCTAGGGATGTC |  |
| CYP707A2 A2-1 R | CTTCATCATATCTTGGACCT |  |
| CYP707A2 A2-2 F | AAGTGTAGTGTGGGGTTAGC |  |
| CYP707A2 A2-2 R | CGCAGTACTATTTATGTGGT |  |
| TUBULIN4-F | CGAGAGGATCACAGCAATACAG |  |
| TUBULIN4-R | GGATCCATTCCACAAAGTAGGA |  |
| HDA9 q-RT F | GGAGAATGTAGCTCGTTGTTGG | qRT-PCR analysis |
| HDA9 q-RT R | GTGCAGATTTTGGAGAATTTGC |  |
| CYP707A1 qRT F | TCATCTCACCACCAAGTA |  |
| CYP707A1 qRT R | AAGGCAATTCTGTCATTCTA |  |
| CYP707A2 qRT F | ATCCATCACTCCTCCGAATTCTTCC |  |
| CYP707A2 qRT R | TCCATTTCCGAATGGCATGTACG |  |
| ACS4 qRT F | ACATGAGACCTCTCCTTAGA |  |
| ACS4 qRT R | CCAGTTAGAGACATTTGACA |  |
| ACS8 qRT F | GGTTTTCCGGCTATCGTTTCA |  |
| ACS8 qRT R | CACACTGCATTATCCGTTACA |  |
| AOX1a qRT F | CTCTTCGTTGGCCTACCGATT |  |
| AOX1a qRT R | AACCATTCCAGGTACTGCTGCTAC |  |
| Lhcb1.1 qRT F | TTGAAGGCTACAGAGTCGCAGGAA |  |
| Lhcb1.1 qRT R | ACCAGTGACGATGGCTTGAACG |  |
| TUBULIN8 qRT F | CGTGGATCACAGCAATACAGAGCC |  |
| TUBULIN8 qRT R | CCTCCGCACTTCCACTTCGTCTTC |  |
